# Optimized extraction, odor modulation, and antioxidant and antimicrobial activities of blue essential oil fro*m Artemisia umbrosa*

**DOI:** 10.3389/fpls.2026.1826250

**Published:** 2026-06-10

**Authors:** Ting Fang, Daoran Pang, Yuxin Li, Xiaohan Feng, Yufei Li, Yingying Zhang, Demin Gao

**Affiliations:** 1 School of Pharmacy, Shandong University of Traditional Chinese Medicine (TCM), Jinan, China; 2 Drug Research Institute, Shandong University of TCM, Jinan, China; 3 School of Basic Medicine, Shandong University of TCM, Jinan, China

**Keywords:** antimicrobial, antioxidant, Asteraceae, response surface methodology, silica gel adsorption, volatile constituents

## Abstract

*Artemisia umbrosa* (Besser) Turcz. ex-Verl. (Asteraceae) produces a rare blue essential oil rich in chamazulene, but its industrial application is hindered by low extraction efficiency and undesirable pungent odors. In this study, an integrated strategy combining ultrasound-assisted hydrodistillation (UAH) optimization and post-extraction odor modulation was developed to obtain a high-quality, color-stable blue essential oil with preserved bioactivity. Extraction parameters were optimized using a Box–Behnken response surface design, with optimal conditions identified as a material-to-liquid ratio of 1:13, an extraction time of 3 h 20 min, and 2% NaCl. Sensory quality was subsequently improved by silica gel adsorption (2 g, 60 °C, 60 min), which effectively reduced pungent odors without compromising blue coloration or chamazulene stability. GC–MS analysis identified 36 volatile constituents, with chamazulene accounting for 50.68% of the total composition. The blue essential oil exhibited strong antioxidant activity, with IC_50_ values of 8.71 μL mL^−1^ (DPPH) and 8.86 μL mL^−1^ (ABTS), outperforming *Artemisia argyi* H. Lév. & Vaniot yellow essential oil. Pronounced antimicrobial activity was observed against *Staphylococcus aureus* (MIC/MBC 21.16/25.94 μL mL^−1^) and *Escherichia coli* (25.94/30.64 μL mL^−1^), and these activities were retained after deodorization. Stability tests indicated that chamazulene is sensitive to heat, light, and oxidative conditions. Overall, this study provides a practical and scalable approach for producing deodorized, chamazulene-rich blue essential oil, supporting its potential application in natural preservation and cosmetic formulations.

## 1 Introduction

Blue essential oils have attracted increasing interest due to their distinctive coloration, unique chemical profiles, and diverse biological activities (Bahr et al., 2018). The characteristic blue hue is primarily attributed to chamazulene, a sesquiterpene derivative formed through the thermal degradation of matricin during distillation in several Asteraceae species, including *Matricaria chamomilla* L. and *Tanacetum annuum* L (Mao et al., 2016; Ettakifi et al., 2023). Chamazulene has been widely reported to exhibit potent antioxidant, anti-inflammatory, and antimicrobial activities, making chamazulene-rich essential oils valuable natural resources for industrial, cosmetic, and functional applications (Huang et al., 2022; Belcadi et al., 2023). In addition, coexisting volatile components such as eucalyptol further contribute to both bioactivity and sensory properties (de Oliveira et al., 2019).

Despite these advantages, the practical utilization of blue essential oils remains constrained by several technical challenges. Conventional hydrodistillation or supercritical CO_2_ extraction often leads to suboptimal yields and partial degradation of thermolabile constituents (Sadeh et al., 2019; Li et al., 2022; Perović et al., 2024; Xu et al., 2026).

Recent advances in ultrasound-assisted hydrodistillation (UAH), particularly when combined with response surface methodology (RSM), have demonstrated improved extraction efficiency and reproducibility while mitigating thermal damage (Wang et al., 2012; Addo et al., 2022). The underlying principle of UAH enhancement lies in acoustic cavitation: the ultrasonic waves generate, expand, and violently collapse microbubbles within the medium. This cavitation effect creates intense local shear forces and micro-jetting that physically disrupt cell walls and glandular structures, thereby facilitating the release of essential oils from plant matrices under milder conditions (Thilakarathna et al., 2023). However, most existing studies focus primarily on yield enhancement or compositional profiling, with limited attention to post-extraction quality refinement and practical applicability (Huang et al., 2021).

Another critical limitation lies in sensory acceptability. Although the intense blue coloration enhances visual appeal, blue essential oils frequently possess pungent, resinous, or irritating odors that restrict their application in aromatherapy, cosmetic formulations, and natural preservation systems (Fisher, 1998; Al-Mijalli et al., 2022). Current strategies for odor reduction—such as solvent treatment or encapsulation—often compromise chromatic stability or bioactive retention (Sharmeen et al., 2021). Consequently, simple and scalable approaches that selectively modulate odor while preserving color, chemical composition, and biological activity remain insufficiently explored.

*Artemisia umbrosa* (Besser) Turcz. Ex-Verl., a perennial herb widely distributed in East Asia, represents a promising and underutilized source of chamazulene-rich blue essential oil. The raw material consists of the aerial parts (herb), typically collected at the beginning of the flowering stage, when the essential oil content and chamazulene accumulation are reported to be highest (Russo et al., 2020). Although *A. umbrosa* is not yet a pharmacopoeial raw material, related species such as *A. vulgaris* and *A. absinthium* are official in several European and Asian pharmacopoeias, with standardization based on thujone, eucalyptol, or total essential oil content. Ethnopharmacological records document its traditional use in menstrual regulation, rheumatism relief, and insect repellence (Guo et al., 2019; Ekiert et al., 2020; Luo et al., 2022). Notably, recent chemical analyses have revealed chamazulene contents exceeding 50%, markedly higher than those reported for most other blue essential oil sources (Rizvi et al., 2018; Mailänder et al., 2022). These characteristics highlight *A. umbrosa* as an attractive candidate for the development of standardized, high-quality blue essential oils with industrial and functional value (Zhou et al., 2022).

However, current research on *A. umbrosa* blue essential oil remains fragmented. Extraction processes have rarely been systematically optimized using statistical approaches such as RSM, and post-extraction sensory modulation strategies are largely unexplored. More importantly, integrated evaluations that simultaneously address extraction efficiency, odor control, chemical stability, and bioactivity are scarce in the existing literature (Laribi et al., 2015; Yuan et al., 2025).

Distinct from previous studies, the present work integrates extraction optimization, post-extraction sensory modulation, and bioactivity–stability evaluation into a unified framework. Specifically, this study aims to: (i) optimize ultrasound-assisted hydrodistillation of *A. umbrosa* blue essential oil using a Box–Behnken design; (ii) improve sensory properties through silica gel adsorption while preserving chamazulene content and color stability; (iii) characterize the chemical composition via GC–MS analysis; (iv) evaluate antioxidant and antimicrobial activities; and (v) assess the stability of key constituents under thermal, oxidative, and photolytic conditions. Collectively, these findings are intended to establish a practical and scalable strategy for the industrial utilization of chamazulene-rich blue essential oil from *A. umbrosa*.

## 2 Methods

### 2.1 Plant materials

Fresh aerial parts of *Artemisia umbrosa* (Besser) Turcz. ex-Verl. were harvested during the peak flowering stage from Shandong Rende Zhi Ai Biotechnology Co., Ltd (Shandong, China) (117°46’20.58”E, 36°35’46.86”N; altitude: 399 m). The plants were collected in August 2025 at the beginning of the flowering stage from their natural habitat, as this species is not yet commercially cultivated. Botanical identification was conducted by Prof. Xu Lingchuan (School of Pharmacy, Shandong University of Traditional Chinese Medicine), and a voucher specimen (SDTCM-YA001) was deposited in the university herbarium. Plant material was shade-dried at 25 ± 2 °C for 72 h before use.

For comparative purposes, the yellow essential oil of *Artemisia argyi* H. Lév. & Vaniot was purchased from Shandong Rende Zhi Ai Biotechnology Co., Ltd. (the same supplier that provided the raw material of *A. umbrosa*). The oil was obtained by steam distillation. A voucher specimen (SDTCM-ARG001) was deposited in the herbarium of Shandong University of Traditional Chinese Medicine.

### 2.2 Optimization of extraction process

#### 2.2.1 Single-factor experiments

Ultrasound-assisted hydrodistillation (UAH) was performed using an integrated system consisting of an ultrasonic cleaner (KQ-500DE, 40 kHz, maximum power 500 W, adjustable, power density 0.12 W/mL, Shandong Chuangjia Instrument Co., Ltd., China) coupled in series with a conventional Clevenger-type apparatus (Shandong Jieshikai Biotechnology Co., Ltd., China). The process comprised two sequential stages: (1) Ultrasonic Pretreatment: The plant material (fresh or dried) was immersed in distilled water (with NaCl) within the ultrasonic chamber. It was subjected to ultrasonication at a fixed power of 100 W for a specified duration (0–45 min) at ambient temperature to disrupt cellular structures. (2) Immediate Hydrodistillation: Immediately following ultrasonication, the entire mixture (plant material and solvent) was transferred without delay to the connected Clevenger apparatus, where conventional hydrodistillation commenced and continued for the designated total extraction time (2–4 h). This configuration ensures that the cavitation effect is applied as a pretreatment to enhance the subsequent distillation efficiency. Accordingly, The following factors were assessed during the Ultrasonic Pretreatment and Hydrodistillation stages: plant material state (fresh vs. dried), total extraction time (2–4 h, which encompasses both the ultrasonication duration and the subsequent pure distillation time), ultrasonication pre-treatment duration (0–45 min), material-to-liquid ratio (1:8–1:16 w/v), salinity (0–4% NaCl, w/v), and particle size (20 mesh, 40 mesh, chopped). Each parameter was varied independently while keeping other conditions constant. The essential oil content (mL/100 g dry weight) obtained from the raw material under each set of conditions was recorded, and all experiments were conducted in triplicate (Madhumita et al., 2019).

#### 2.2.2 Response surface methodology

Based on single-factor results, a Box–Behnken design (BBD) was implemented using Design-Expert 13.0 software. The distillate dropping rate was controlled at 2–3 s per drop, and the essential oil was dried over anhydrous sodium sulfate. Three variables were examined: salinity (A, 1–4%), material-to-liquid ratio (B, 1:10–1:16), and extraction time (C, 2–4 h). Each factor was studied at three levels, with experiments randomized to reduce systematic bias. Analysis of variance (ANOVA) was performed to evaluate model significance, and three-dimensional response surface plots were generated (Li et al., 2024; Wang et al., 2025; Zhao et al., 2025).

### 2.3 Flavor modification and color retention

#### 2.3.1 Chamazulene isolation and purification

Chamazulene (Beijing INOKAI Technology Co., Ltd., China) was used as a reference standard. Chamazulene was isolated by preparative HPLC on a COSMOSIL PBr column (10 × 250 mm, 5 μm, Shanxi Nuotai Biotechnology Co., Ltd., China) using acetonitrile-water (95:5, v/v) as the mobile phase at 1.0 mL/min. Essential oil (1 mg/mL in acetonitrile) was injected (100 μL) and monitored at 254 nm. The chamazulene fraction was consistently eluted at 17.75 ± 0.25 min, collected manually, concentrated under reduced pressure (100–116 mbar, 39 °C). The obtained product was stored at 4 °C prior to further analysis. The purity of isolated chamazulene (> 90%) was verified by analytical HPLC, UV–Vis spectroscopy (λ_max = 336 nm), and GC–MS analysis. The purified chamazulene was subsequently subjected to NMR analysis for structural confirmation, as described below.

#### 2.3.2 Silica gel adsorption optimization

The deodorization of the crude blue essential oil was achieved through a silica gel adsorption process, which was carried out in three sequential stages: (1) Pretreatment: Commercial silica gel (200–300 mesh, Shanghai Haohong Biomedical Technology Co., Ltd., China) was pre-activated by heating at 120 °C for 2 h prior to use. A specified dosage (1–4 g per 15 mL of oil) of the activated silica gel was weighed. (2) Ultrasound-Assisted Adsorption: The essential oil was mixed with the pre-weighed silica gel in a sealed glass vessel. This adsorption step was conducted under ultrasonic irradiation using an ultrasonic bath (parameters: frequency 40 kHz, power 100 W, Shandong Chuangjia Instrument Co., Ltd., China). The mixture was subjected to ultrasonic treatment at a controlled temperature (varied between 40–80 °C according to the orthogonal design) for a specified duration (30–90 min). The ultrasonic agitation was employed to enhance the mass transfer and interaction between the adsorbent and the undesirable odor-causing compounds (e.g., ketones). (3) Separation and Recovery: Following adsorption, the mixture was immediately filtered under vacuum through Whatman No. 1 filter paper (Shanghai Haohong Biomedical Technology Co., Ltd., China) to separate the spent silica gel from the deodorized essential oil. The filtrate (deodorized oil) was collected in amber vials and stored at 4 °C for subsequent analysis. To optimize this process, a three-factor, three-level orthogonal experimental design was employed to evaluate the effects of silica gel dosage, adsorption temperature, and contact time on deodorization efficiency. Based on GC-MS profiling and literature reports on *Artemisia* essential oils, eucalyptol and (+)-2-bornanone were selected as representative chemical markers for pungent odor. Their relative contents (%) in the essential oil before and after silica gel adsorption were determined by GC-MS (Agilent 7890/5977B, EI mode 70 eV, Shanghai, China). The effectiveness of the treatment was evaluated based on color retention, measured spectrophotometrically (reported as ΔE values), and the stability of the key component chamazulene, as determined by GC analysis (Shih et al., 2009).

Sensory evaluation was performed by a panel of 10 trained assessors (2-hour training). Samples were coded with random three-digit numbers and presented in sealed amber vials at room temperature. Odor intensity/pungency was scored using a 10-point scale, where 0 indicated no pungent odor and 10 indicated extremely strong pungent odor. Each sample was evaluated in triplicate, and the mean odor score was used to calculate odor reduction. Panelists were blinded to treatment conditions.

### 2.4 NMR analysis of isolated chamazulene

The chemical structure of the isolated chamazulene obtained in Section 2.3.1 was confirmed by one-dimensional ^1^H and ^13^C NMR spectroscopy. Approximately 50 mg of purified chamazulene was accurately weighed, dissolved in CD_2_Cl_2_ (Beijing INOKAI Technology Co., Ltd., China), and transferred to a 5 mm NMR tube. ^1^H NMR and ^13^C NMR spectra were recorded on an NMR spectrometer operating at 600 MHz for ^1^H and 151 MHz for ^13^C, respectively. Chemical shifts (δ) were reported in parts per million (ppm) relative to the residual solvent signals of CD_2_Cl_2_. The acquired NMR data were used to confirm the characteristic azulene framework and substituent pattern of chamazulene.

### 2.5 Compositional analysis

Essential oil composition was analyzed using GC–MS. Chromatographic separation was carried out on an HP-5MS capillary column (30 m × 0.25 mm × 0.25 μm, Agilent Technologies, Shanghai, China) using helium as carrier gas at a constant flow of 1.0 mL min^−1^. The oven temperature program started at 50 °C (held for 2 min), increased to 120 °C at 5 °C min^−1^ (held for 2 min), and then ramped to 250 °C at 10 °C min^−1^ (held for 2 min). A 1 μL aliquot was injected in split mode (10:1 split ratio). Analytes were tentatively identified by matching their mass spectra against the NIST 17 database (≥ 85% match) and confirmed by co-injection with authentic reference compounds (Guan et al., 2019). It should be noted that, among the identified compounds, only chamazulene was confirmed by co-injection with an authentic reference standard. The identification of all other compounds is therefore considered tentative, based on spectral matching and retention index consistency.

Quantitative analysis of the essential oil components was performed using the peak area normalization method. The relative content of each identified compound was expressed as a percentage of the total peak area in the total ion chromatogram (TIC). All samples were analyzed in triplicate, and the results are reported as mean ± standard deviation (SD).

### 2.6 Bioactivity assays

#### 2.6.1 Antioxidant activity

DPPH and ABTS (Biosharp Life Sciences, Beijing, China) assays were performed following standardized methods. DPPH assay: 1 mL of each sample concentration was combined with 2 mL ethanolic 0.1 mM DPPH, kept in darkness for 30 min, then read at 517 nm (Kayahan and Saloglu, 2021). ABTS assay: the radical cation reagent was prepared and adjusted to an absorbance of 0.70 ± 0.02 at 734 nm. A 1 mL aliquot of sample was mixed with 2 mL of this ABTS solution, allowed to react for 6 min, and the absorbance was recorded at 734 nm. To account for the potential interference of the intrinsic blue color of the essential oil and chamazulene at 734 nm, sample blanks containing the same concentration of the test substance without the ABTS reagent were prepared, and their absorbance values were subtracted from the corresponding test readings. Vitamin C (Vc) served as the reference standard. IC_50_ values were calculated using GraphPad Prism 9.0 (Gharsallah et al., 2022; Kačániová et al., 2023).

#### 2.6.2 Antimicrobial activity

Antimicrobial activity was tested against Gram-positive (*Staphylococcus aureus* ATCC 25923, MRSA ATCC 43300), Gram-negative *(Escherichia coli* ATCC 25922, *Pseudomonas aeruginosa* ATCC 27853, *Proteus mirabilis* ATCC 12453), and fungal (*Candida albicans* ATCC 10231) strains. All the above-mentioned strains were provided by the Microbiology Teaching and Research Section, Shandong University of Traditional Chinese Medicine (Shandong, China). Cultures were standardized to 0.5 McFarland (5×10^5^–5×10^6^ CFU/mL). Essential oils were dissolved in 0.1% Tween-80 (Bioss, Beijing, China), sterilized by 0.22 μm filtration, and serially diluted (100–1.56 μL/mL). Gentamicin sulfate at a concentration of 50 μg/mL was employed as a positive control. MIC and MBC were determined using broth microdilution according to CLSI guidelines (Cazella et al., 2019).

### 2.7 Stability tests

The stability of the samples was systematically evaluated by exposing them to a variety of storage environments: (a) Thermal stability: 4 °C, 25 °C, 50 °C for 30 days; (b) Oxidative stability: sealed vs. open-air at 25 °C; (c) Photostability: light vs. dark at 25 °C. Chamazulene and eucalyptol contents were quantified at defined intervals using GC–MS (Mahdi et al., 2021; Gonciarz et al., 2025).

### 2.8 Statistical analysis

All experiments were performed in triplicate. Data were expressed as mean ± standard deviation (SD). One-way ANOVA followed by Tukey’s multiple comparison test was applied. Differences were considered significant at *p* < 0.05. Statistical analyses were performed with SPSS 26.0 (IBM, Armonk, NY, USA).

## 3 Results

### 3.1 Optimization of UAH extraction

Single-factor experiments indicated that the essential oil content obtained from the raw materialwas significantly influenced by plant material state, extraction time, ultrasonication duration, material-to-liquid ratio, salinity, and particle size. Fresh aerial parts consistently yielded higher oil recovery than dried material (**Supplementary Table 1**). The recovered oil content increased with extraction time up to 3 h and ultrasonication duration up to 30 min, after which no further significant improvement was observed (*p* > 0.05). Increasing the material-to-liquid ratio enhanced extraction efficiency, with an optimal ratio of 1:12, resulting in an 18.5% increase in recovered oil content compared with the lowest ratio tested. Salinity improved extraction efficiency up to 3%, while blade-cut plant material produced the highest oil recovery among particle size treatments.

**Figure 1:**
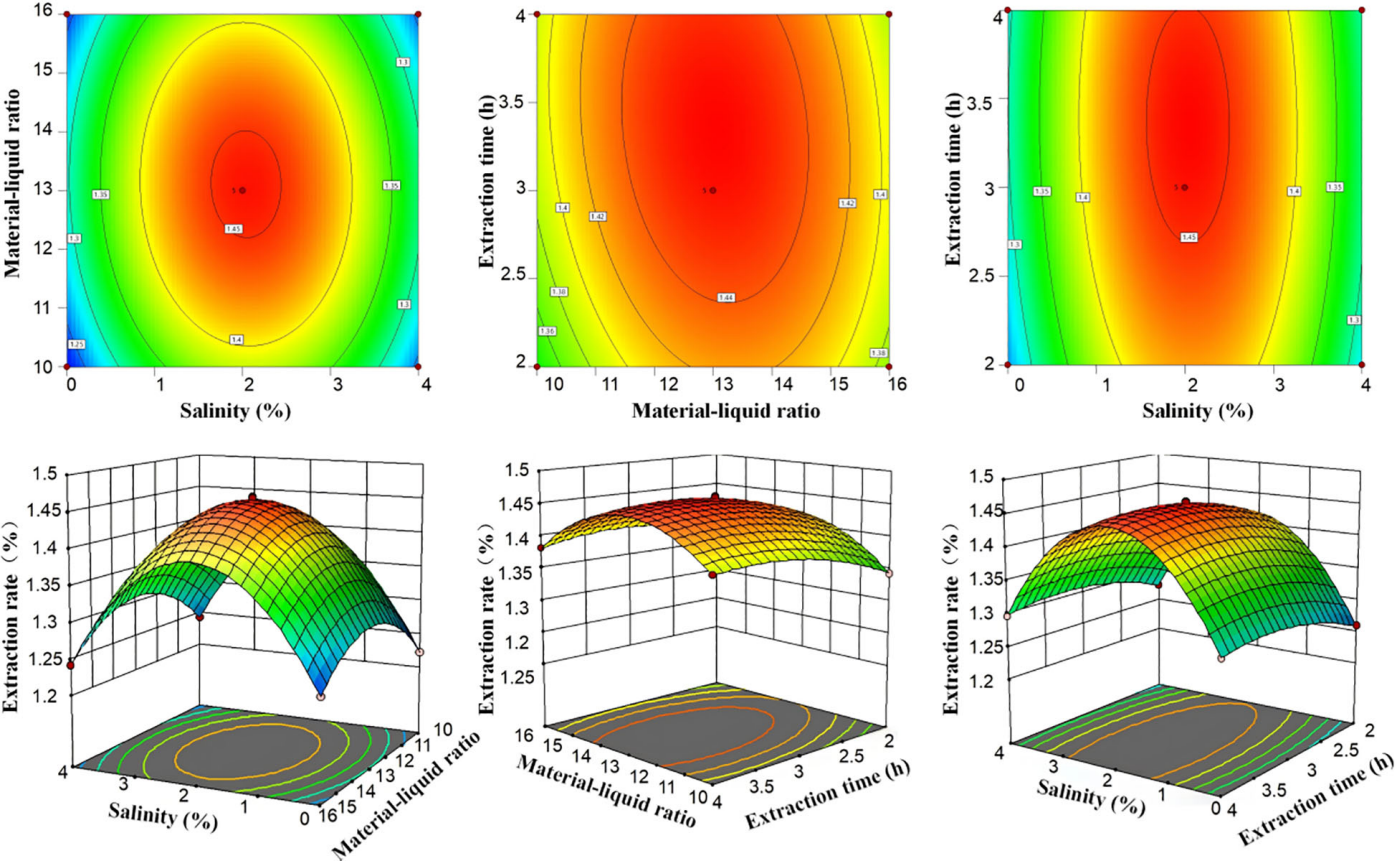
Contour and response surface plots.

Based on these results, a Box–Behnken design was applied to optimize the extraction process. The quadratic regression model was highly significant (*p* < 0.01) with a strong coefficient of determination (R² = 0.957), indicating good model fitness (**Tables 1**, **2**). Response surface analysis revealed significant interactive effects among salinity, material-to-liquid ratio, and extraction time (**Figure 1**). The optimal extraction conditions were identified as 2.0% salinity, a material-to-liquid ratio of 1:13, and an extraction time of 3 h 20 min. Under these conditions, the predicted essential oil content obtained from the raw material (1.46 ± 0.02%, v/w) was experimentally validated, with a relative deviation of only 2.7%, confirming the reliability of the model.

**Table 1:** Response surface experiment results of blue essential oil of *Artemisia umbrosa*.

No.	A (%)	B	C (h)	Extraction rate (M ± SD%)
1	0	1:10	3	1.22 ± 0.02
2	0	1:13	2	1.26 ± 0.02
3	0	1:13	4	1.29 ± 0.02
4	0	1:16	3	1.24 ± 0.00
5	2	1:10	2	1.34 ± 0.04
6	2	1:10	4	1.39 ± 0.05
7	2	1:13	3	1.46 ± 0.03
8	2	1:13	3	1.45 ± 0.02
9	2	1:13	3	1.45 ± 0.04
10	2	1:13	3	1.46 ± 0.06
11	2	1:13	3	1.46 ± 0.07
12	2	1:13	4	1.38 ± 0.05
13	2	1:16	2	1.37 ± 0.00
14	4	1:10	3	1.24 ± 0.02
15	4	1:13	2	1.28 ± 0.07
16	4	1:13	4	1.30 ± 0.05
17	4	1:16	3	1.24 ± 0.02

A, Salinity; B, Material - to - liquid ratio; C, Extraction time. Data are reported as the M ± SD% (*n* = 3).

**Table 2:** Analysis of variance (ANOVA) for the response surface regression model of essential oil extraction optimization.

Factor	Square sum	Degrees of freedom	Mean square value	*F*-value	*P*-value
Mold	0.1306	9	0.0145	700.58	< 0.0001
A	0.0003	1	0.0003	15.09	0.0060
B	0.0002	1	0.0002	9.66	0.0171
C	0.0015	1	0.0015	73.02	< 0.0001
AB	0.0001	1	0.0001	4.83	0.0640
AC	0.0000	1	0.0000	1.21	0.3083
BC	0.0004	1	0.0004	19.31	0.0032
A^2^	0.1002	1	0.1002	4836.34	< 0.0001
B^2^	0.0188	1	0.0188	905.67	< 0.0001
C^2^	0.0016	1	0.0016	75.32	< 0.0001
Inaccuracies	0.0001	7	0.0000		
Lost proposal	0.0000	3	8.333 × 10^−6^	0.2778	0.8395
Pure error	0.0001	4	0.0000		
Aggregate difference	0.1308	16			
R^2^ = 0.9989		R2 adj = 0.9975		R2 pre = 0.9955	

A, Salinity; B, Material–liquid ratio; C, Extraction time. *P* < 0.01, highly significant difference; *P* < 0.05, significant difference.

### 3.2 Flavor modification and color retention

Through sensory observation, orthogonal analysis identified 2 g/15 mL, 60 °C, and 60 min as the optimal treatment, resulting in a 42.3% reduction in odor score, > 98% chamazulene retention, and ΔE < 1.0 in colorimetric evaluation (**Table 3**). Silica gel adsorption significantly reduced pungent odors while preserving the characteristic blue coloration of the essential oil. Increasing silica dosage enhanced odor removal but resulted in a gradual decrease in color intensity, whereas temperatures above 60 °C caused eucalyptol degradation (*p* < 0.05) (**Table 4**).

**Table 3:** ANOVA and orthogonal analysis of color preservation after silica gel adsorption.

No.	A (g)	B (°C)	C (min)	Absorbance (M ± SD)					Chamazulene content (M ± SD %)					Eucalyptol and (+)-2-Bornanone content (M ± SD%)				
1	1	20	30	6.60 ± 0.01					6.60 ± 0.05					29.01 ± 0.23				
2	1	40	90	6.66 ± 0.03					6.66 ± 0.03					28.73 ± 0.10				
3	1	80	60	6.75 ± 0.00					6.78 ± 0.05					28.51 ± 0.10				
4	2	20	60	6.61 ± 0.00					6.61 ± 0.00					28.09 ± 0.20				
5	2	40	30	6.70 ± 0.05					6.70 ± 0.04					27.84 ± 0.20				
6	2	80	90	6.66 ± 0.06					6.66 ± 0.06					28.37 ± 0.28				
7	4	20	90	6.62 ± 0.01					6.62 ± 0.06					28.64 ± 0.22				
8	4	40	60	6.84 ± 0.03					6.84 ± 0.06					27.89 ± 0.23				
9	4	80	30	6.91 ± 0.02					6.91 ± 0.05					28.01 ± 0.23				
K_1_				0.54		0.51		0.53	6.67		6.61		6.74	28.75		28.58		28.29
K_2_				0.53		0.53		0.52	6.66		6.73		6.73	28.10		28.15		28.16
K_3_				0.52		0.54		0.53	6.79		6.77		6.65	28.18		28.30		28.57
R				0.02		0.03		0.01	0.13		0.16		0.09	0.65		0.43		0.41
Ability to influence				B>A>C					B>A>C					A>B>C				
Best combination				A_1_B_3_C_1_					A_3_B_3_C_1_					A_2_B_2_C_2_				
Experimental factors				SS	df	MS	F	*P*	SS	df	MS	F	*P*	SS	df	MS	F	*P*
A				0.002	2	0.001	1.794	0.192	0.096	2	0.048	3.311	0.057	2.266	2	1.133	7.726	0.003
B				0.004	2	0.002	4.094	0.032	0.143	2	0.071	4.946	0.018	0.833	2	0.416	2.838	0.082
C				0.000	2	0.000	0.333	0.720	0.051	2	0.026	1.768	0.196	0.814	2	0.407	2.775	0.086
Inaccuracies				0.010	20	0.001			0.289	20	0.014			2.933	20	0.147		
Total				7.534	27				1215.961	27				21696.582	27			

A, Silica gel dosage (g); B, Adsorption temperature (°C); C, Adsorption time (min); SS, sum of squares; df, degrees of freedom; MS, mean square; F, statistic; *P*, probability; N, sample size. Data are presented as M ± SD% (*n* = 3). Statistical analysis was conducted using one-way ANOVA.

**Table 4:** Compositional analysis of *Artemisia umbrosa* blue essential oils under different treatments and *Artemisia argyi* yellow essential oil.

No.	RT	RI	Compounds	Molecular formula	Molecular weight	Relative content (M ± SD%)											
						YAL	AL	Tr1	Tr2	Tr3	Tr4	Tr5	Tr6	Tr7	Tr8	Tr9	Tr10
1	6.00	907.2	Santolina triene	C10H16	136	–	0.21 ± 0.02	–	–	–	–	–	–	–	–	–	–
2	6.54	964.5	1,8-Cineole	C10H16	136	–	0.37 ± 0.01	0.85 ± 0.02	0.87 ± 0.01	0.87 ± 0.01	0.88 ± 0.01	0.89 ± 0.01	0.89 ± 0.01	0.90 ± 0.02	0.89 ± 0.01	0.88 ± 0.03	0.85 ± 0.03
3	7.10	1026.8	Camphene	C10H16	136	–	1.63 ± 0.04	1.12 ± 0.01	1.18 ± 0.01	1.17 ± 0.03	1.21 ± 0.02	1.22 ± 0.01	1.20 ± 0.03	1.19 ± 0.01	1.21 ± 0.02	1.21 ± 0.02	1.19 ± 0.02
4	7.78	1098.3	Sabinene	C10H16	136	0.57 ± 0.03	1.95 ± 0.04	3.52 ± 0.06	3.55 ± 0.02	3.58 ± 0.02	3.65 ± 0.04	3.64 ± 0.03	3.65 ± 0.07	3.71 ± 0.04	3.74 ± 0.02	3.68 ± 0.04	3.56 ± 0.03
5	7.86	1108.5	*α*-Thujene	C10H16	136	–	0.94 ± 0.02	0.99 ± 0.00	1.04 ± 0.01	1.04 ± 0.02	1.03 ± 0.01	1.04 ± 0.01	1.03 ± 0.02	1.06 ± 0.02	1.07 ± 0.01	1.04 ± 0.02	1.04 ± 0.01
6	7.93	980.5	1-Octen-3-ol	C_8_H_16_O	128	–	0.52 ± 0.01	–	–	–	–	–	–	–	–	–	–
7	8.27	990.7	*β*-Myrcene	C10H16	136	1.00 ± 0.05	0.38 ± 0.01	2.44 ± 0.07	2.55 ± 0.02	2.52 ± 0.04	2.59 ± 0.04	2.60 ± 0.05	2.55 ± 0.04	2.57 ± 0.12	2.63 ± 0.05	2.58 ± 0.04	2.54 ± 0.05
8	8.63	1025.9	*α*-Phellandrene	C10H16	136	0.36 ± 0.02	4.09 ± 0.04	1.38 ± 0.05	1.47 ± 0.05	1.40 ± 0.06	1.47 ± 0.06	1.47 ± 0.07	1.45 ± 0.10	1.48 ± 0.06	1.49 ± 0.06	1.43 ± 0.03	1.47 ± 0.09
9	8.98	1053.6	*p*-*α*-Phellandrene	C10H16	136	1.01 ± 0.05	1.01 ± 0.00	3.08 ± 0.02	3.14 ± 0.05	3.19 ± 0.04	3.25 ± 0.04	3.24 ± 0.03	3.29 ± 0.07	3.2 ± 0.02	3.26 ± 0.02	3.22 ± 0.01	3.18 ± 0.02
10	9.13	1066.8	Terpinolene	C10H18	138	0.46 ± 0.02	–	–	–	–	–	–	–	–	–	–	–
11	9.21	1058.7	*o*-Cymene	C10H14	134	0.44 ± 0.02	2.24 ± 0.05	1.78 ± 0.01	1.82 ± 0.05	1.88 ± 0.02	1.88 ± 0.03	1.9 ± 0.02	1.90 ± 0.02	1.79 ± 0.04	1.9 ± 0.02	1.88 ± 0.01	1.84 ± 0.00
12	9.33	1070.5	D-limonene	C10H16	136	–	0.69 ± 0.03	1.11 ± 0.02	1.09 ± 0.02	1.10 ± 0.01	1.09 ± 0.02	1.08 ± 0.02	1.08 ± 0.04	1.05 ± 0.04	1.09 ± 0.02	1.09 ± 0.01	1.06 ± 0.02
13	9.40	1032.0	Eucalyptol	C10H18O	154	8.88 ± 0.49	16.61 ± 0.34	27.06 ± 0.26	26.73 ± 0.23	26.66 ± 0.11	26.18 ± 0.27	25.94 ± 0.22	26.52 ± 0.40	26.81 ± 0.49	26.06 ± 0.26	26.21 ± 0.49	26.69 ± 0.16
14	10.20	1069.6	*γ*-Terpinene	C10H16	136	1.66 ± 0.08	1.43 ± 0.05	4.30 ± 0.03	4.33 ± 0.02	4.41 ± 0.01	4.45 ± 0.03	4.45 ± 0.01	4.42 ± 0.05	4.41 ± 0.01	4.54 ± 0.02	4.48 ± 0.04	4.34 ± 0.02
15	10.45	1074.8	Sabinol	C10H18O	154	–	1.26 ± 0.03	1.85 ± 0.07	1.83 ± 0.10	1.84 ± 0.02	1.90 ± 0.03	1.82 ± 0.02	1.8 ± 0.05	1.73 ± 0.01	1.73 ± 0.02	1.73 ± 0.06	1.86 ± 0.04
16	11.05	1134.6	*p*-Mentha-1,3-diene	C10H16	136	0.37 ± 0.00	0.66 ± 0.02	0.99 ± 0.01	1.02 ± 0.02	1.01 ± 0.02	1.01 ± 0.02	1.04 ± 0.01	1.04 ± 0.02	1.02 ± 0.00	1.04 ± 0.01	1.04 ± 0.02	1.02 ± 0.01
17	11.35	1154.7	(–)-Borneol	C10H18O	154	–	0.79 ± 0.03	0.51 ± 0.00	0.51 ± 0.01	0.48 ± 0.01	0.48 ± 0.01	0.49 ± 0.00	0.48 ± 0.02	0.46 ± 0.02	0.43 ± 0.01	0.44 ± 0.01	0.51 ± 0.00
18	11.40	1098.8	Linalool	C10H18O	154	0.28 ± 0.10	–	1.00 ± 0.00	1.02 ± 0.01	0.99 ± 0.02	1.02 ± 0.02	1.04 ± 0.01	1.02 ± 0.03	0.96 ± 0.03	0.98 ± 0.02	0.98 ± 0.02	1.03 ± 0.01
19	11.50	1107.9	*α*-Vetivone	C10H14O	150	0.30 ± 0.12	–	0.41 ± 0.02	0.42 ± 0.00	0.42 ± 0.02	0.42 ± 0.01	0.43 ± 0.01	0.43 ± 0.00	0.44 ± 0.02	0.43 ± 0.00	0.43 ± 0.02	0.45 ± 0.01
20	11.55	1169.5	Thujone	C10H16O	152	–	7.28 ± 0.07	1.03 ± 0.02	1.06 ± 0.01	1.04 ± 0.03	1.04 ± 0.03	1.07 ± 0.00	1.07 ± 0.01	1.26 ± 0.02	1.13 ± 0.02	1.34 ± 0.01	1.27 ± 0.01
21	11.87	1197.6	(–)-Fenchone	C10H16O	152	–	1.51 ± 0.04	–	–	–	–	–	–	–	–	–	–
22	12.02	1211.8	Dihydrocarveol	C10H18O	154	–	0.65 ± 0.02	0.68 ± 0.01	0.69 ± 0.00	0.66 ± 0.01	0.67 ± 0.01	0.22 ± 0.01	0.33 ± 0.01	0.63 ± 0.01	0.63 ± 0.01	0.64 ± 0.01	0.69 ± 0.01
23	12.11	1229.7	Verbenone	C10H14O	150	–	–	1.27 ± 0.02	1.28 ± 0.02	1.25 ± 0.03	1.23 ± 0.01	0.69 ± 0.00	0.67 ± 0.02	1.23 ± 0.04	1.23 ± 0.02	1.26 ± 0.02	1.30 ± 0.03
24	12.54	1257.5	cis-Sabinol	C10H16O	152	–	0.72 ± 0.04	0.52 ± 0.03	0.52 ± 0.01	0.51 ± 0.01	0.51 ± 0.02	1.29 ± 0.00	1.26 ± 0.03	0.48 ± 0.02	0.47 ± 0.01	0.46 ± 0.01	0.52 ± 0.01
25	12.66	1143.8	(+)-2-Bornanone	C10H16O	152	1.88 ± 0.08	5.66 ± 0.29	5.03 ± 0.09	5.07 ± 0.02	5.07 ± 0.02	5.04 ± 0.06	0.51 ± 0.02	0.51 ± 0.01	5.06 ± 0.06	5.01 ± 0.04	4.96 ± 0.02	5.15 ± 0.03
26	13.22	1307.6	cis-Chrysanthenol	C10H16O	152	–	1.52 ± 0.08	0.50 ± 0.02	0.48 ± 0.01	0.47 ± 0.01	0.49 ± 0.01	5.03 ± 0.10	4.98 ± 0.03	0.45 ± 0.01	0.47 ± 0.00	0.45 ± 0.01	0.43 ± 0.03
27	13.30	1315.8	endo-Borneol	C10H18O	154	0.76 ± 0.03	6.72 ± 0.28	1.95 ± 0.04	1.89 ± 0.02	1.85 ± 0.01	1.91 ± 0.03	0.48 ± 0.02	0.47 ± 0.00	1.82 ± 0.04	1.83 ± 0.04	1.80 ± 0.04	1.88 ± 0.05
28	13.51	1334.7	*(S)*-Lavandulyl acetate	C_12_H_20_O_2_	196	–	6.78 ± 0.33	–	–	–	–	–	–	–	–	–	–
29	13.63	1346.9	*(R)*-(–)-*α*-Terpineol	C10H18O	154	3.80 ± 0.09	3.32 ± 0.15	5.69 ± 0.07	5.56 ± 0.06	5.52 ± 0.04	5.64 ± 0.02	1.90 ± 0.05	1.85 ± 0.03	5.52 ± 0.07	5.57 ± 0.09	5.47 ± 0.04	5.65 ± 0.03
30	14.02	1382.5	*α*-Terpineol	C10H18O	154	1.57 ± 0.07	1.79 ± 0.06	2.92 ± 0.05	2.85 ± 0.06	–	2.89 ± 0.03	5.68 ± 0.11	5.55 ± 0.04	2.72 ± 0.04	2.70 ± 0.04	2.67 ± 0.08	2.90 ± 0.05
31	14.50	1426.7	trans-Sabinol	C10H18O	154	–	–	0.47 ± 0.02	0.47 ± 0.01	2.78 ± 0.08	0.17 ± 0.00	2.91 ± 0.05	2.78 ± 0.05	0.14 ± 0.20	0.5 ± 0.03	0.54 ± 0.01	–
32	14.82	1457.8	trans-Carveol	C10H18O	154	–	1.20 ± 0.08	–	–	–	–	–	–	–	–	–	–
33	16.63	1639.5	Bornyl acetate	C_12_H_20_O_2_	196	–	0.35 ± 0.01	–	–	–	–	–	–	–	–	–	–
34	17.81	1752.6	*γ*-Elemene	C15H24	204	–	–	0.46 ± 0.02	0.45 ± 0.04	0.48 ± 0.02	0.51 ± 0.02	0.52 ± 0.02	0.48 ± 0.02	0.48 ± 0.03	0.42 ± 0.01	0.43 ± 0.02	0.50 ± 0.02
35	18.60	1828.7	Copaene	C15H24	204	–	–	0.41 ± 0.02	0.14 ± 0.00	0.37 ± 0.03	0.41 ± 0.01	0.42 ± 0.02	0.42 ± 0.02	0.44 ± 0.00	0.18 ± 0.00	–	0.42 ± 0.01
36	18.90	1858.8	(–)-*α*-Copaene	C15H24	204	–	–	–	–	–	–	–	–	–	–	–	0.10 ± 0.00
37	19.41	1417.6	Caryophyllene	C15H24	204	2.59 ± 0.12	7.15 ± 0.41	4.48 ± 0.02	4.48 ± 0.04	4.55 ± 0.00	4.47 ± 0.05	4.46 ± 0.02	4.49 ± 0.06	4.47 ± 0.05	4.53 ± 0.03	4.60 ± 0.09	4.42 ± 0.06
38	19.98	1517.8	Humulene	C15H24	204	0.78 ± 0.02	1.30 ± 0.04	1.28 ± 0.02	1.28 ± 0.00	1.29 ± 0.03	1.28 ± 0.02	1.29 ± 0.01	1.27 ± 0.03	1.28 ± 0.02	1.31 ± 0.02	1.31 ± 0.04	1.25 ± 0.02
39	20.19	1537.9	(–)-cis-Rose oxide	C15H24O	220	0.50 ± 0.06	1.35 ± 0.06	0.31 ± 0.00	0.30 ± 0.00	0.30 ± 0.01	0.29 ± 0.01	0.30 ± 0.01	0.30 ± 0.01	0.31 ± 0.01	0.30 ± 0.01	0.30 ± 0.01	0.30 ± 0.01
40	20.43	1480.7	Germacrene D	C15H24	204	4.05 ± 0.14	–	4.66 ± 0.03	4.66 ± 0.02	4.72 ± 0.00	4.67 ± 0.06	4.66 ± 0.03	4.66 ± 0.07	4.66 ± 0.07	4.78 ± 0.03	4.80 ± 0.10	4.58 ± 0.07
41	20.51	1577.8	*β*-Caryophyllene	C15H24	204	1.47 ± 0.07	0.38 ± 0.02	1.53 ± 0.01	1.52 ± 0.02	1.56 ± 0.01	1.52 ± 0.02	1.52 ± 0.01	1.53 ± 0.01	1.54 ± 0.03	1.57 ± 0.01	1.59 ± 0.03	1.52 ± 0.02
42	20.66	1591.6	Cyclosativene	C15H24	204	1.49 ± 0.10	0.54 ± 0.04	3.16 ± 0.03	3.11 ± 0.03	3.16 ± 0.02	3.12 ± 0.04	3.12 ± 0.04	3.14 ± 0.03	3.23 ± 0.14	3.19 ± 0.02	3.23 ± 0.08	3.09 ± 0.06
43	21.04	1626.8	(+)-Sabinene	C15H24	204	–	–	0.41 ± 0.01	0.39 ± 0.01	0.41 ± 0.02	0.40 ± 0.02	0.40 ± 0.01	0.42 ± 0.02	0.42 ± 0.03	0.42 ± 0.01	0.42 ± 0.02	0.40 ± 0.02
44	21.84	1698.7	(–)-Guaiol	C15H26O	222	1.88 ± 0.08	0.31 ± 0.02	0.46 ± 0.01	0.46 ± 0.00	0.46 ± 0.01	0.46 ± 0.01	0.46 ± 0.01	0.45 ± 0.01	0.44 ± 0.01	0.45 ± 0.01	0.45 ± 0.00	0.46 ± 0.01
45	21.92	1579.8	Caryophyllene oxide	C15H24O	220	1.22 ± 0.09	0.78 ± 0.02	0.30 ± 0.01	0.30 ± 0.00	0.27 ± 0.01	0.29 ± 0.01	0.3 ± 0.01	0.28 ± 0.01	0.29 ± 0.01	0.29 ± 0.01	0.29 ± 0.01	0.30 ± 0.00
46	22.29	1550.7	Diepicedrene-1-oxide	C15H24O	220	0.36 ± 0.07	–	–	–	–	–	–	–	–	–	–	–
47	22.36	1518.8	3,6-Dihydrochamazulene	C14H18	186	0.63 ± 0.09	–	–	–	–	–	–	–	–	–	–	–
48	22.54	1749.6	1-phenyl-2-(2,2,3,3-tetramethylcyclopropylidene)ethene	C14H18	186	0.55 ± 0.03	–	–	–	–	–	–	–	–	–	–	–
49	22.63	1576.8	(–)-Spathulenol	C15H26O	222	0.49 ± 0.03	–	–	–	–	–	–	–	–	–	–	–
50	22.86	1659.7	Neointermedeol	C15H26O	222	2.06 ± 0.1	1.46 ± 0.05	–	–	–	–	–	–	–	–	–	–
51	23.02	1794.8	ledene oxide-(II)	C15H24O	220	0.55 ± 0.05	–	–	–	–	–	–	–	–	–	–	–
52	23.15	1806.7	*α*-Bisabolol	C15H26O	222	0.57 ± 0.05	–	–	–	–	–	–	–	–	–	–	–
53	23.23	1814.8	Humulenol-II	C15H24O	220	0.56 ± 0.05	–	–	–	–	–	–	–	–	–	–	–
54	23.77	1724.6	Chamazulene	C14H16	184	50.68 ± 0.72	–	6.60 ± 0.15	6.66 ± 0.03	6.78 ± 0.05	6.61 ± 0.10	6.70 ± 0.05	6.66 ± 0.06	6.62 ± 0.19	6.84 ± 0.08	6.91 ± 0.09	6.67 ± 0.06
55	24.19	1859.7	(+)-Nootkatol	C17H24O	220	0.36 ± 0.02	–	–	–	–	–	–	–	–	–	–	–
56	24.32	1784.8	Dehydrochamazulene	C14H14	182	1.43 ± 0.08	–	–	–	–	–	–	–	–	–	–	–
Alcohols						20.65	36.87	43.61	43.01	42.22	42.32	47.26	47.49	42.16	41.82	41.84	42.62
Aromatic						0.99	2.24	1.78	1.82	1.88	1.88	1.90	1.90	1.79	1.9	1.88	1.84
Terpenes						71.74	26.37	44.65	44.81	45.43	45.43	45.05	44.92	45.56	46.02	45.79	45.00
Ketones						2.18	12.94	6.47	6.55	6.53	6.50	2.01	2.01	6.76	6.57	6.73	6.87
Alkenes						–	–	–	–	–	–	–	–	–	–	–	0.10
Esters						–	7.13	–	–	–	–	–	–	–	–	–	–
Total						95.56	85.55	96.51	96.19	96.06	96.13	96.22	96.32	96.27	96.31	96.24	96.43

YAL, *A. umbrosa* blue essential oil; AL, *A. argyi* yellow essential oil; Tr1–Tr9, experiment numbers 1–9 of the silica gel adsorption orthogonal experiment, and Tr10, untreated original essential oil. Data are reported as the M ± SD% (*n* = 3). Data are reported as the M ± SD% (n = 3). Experimental linear retention indices (RI) were calculated against a C_7_–C_30_ n-alkane mix (Shanghai Yuanye Bio-Technology Co., Ltd., China) and matched with NIST 17 Chemistry WebBook (± 5-unit tolerance); The table lists the Common Name (or the Systematic Name if no established common name exists) corresponding to each provided systematic chemical name3.3 Chamazulene isolation and purification.

### 3.3 Chamazulene isolation and purification

Chamazulene was successfully isolated from the blue essential oil using preparative HPLC, yielding a product with purity exceeding 90%. The compound consistently eluted at 17.75 ± 0.25 min and was confirmed by GC analysis, UV–Vis spectroscopy (λ_max_ 336 nm), and mass spectrometry (m/z 184 [M]^+^). The optimized isolation procedure enabled efficient recovery of photostable chamazulene for subsequent structural and functional analyses.

### 3.4 Structure elucidation of chamazulene

**Figure 2:**
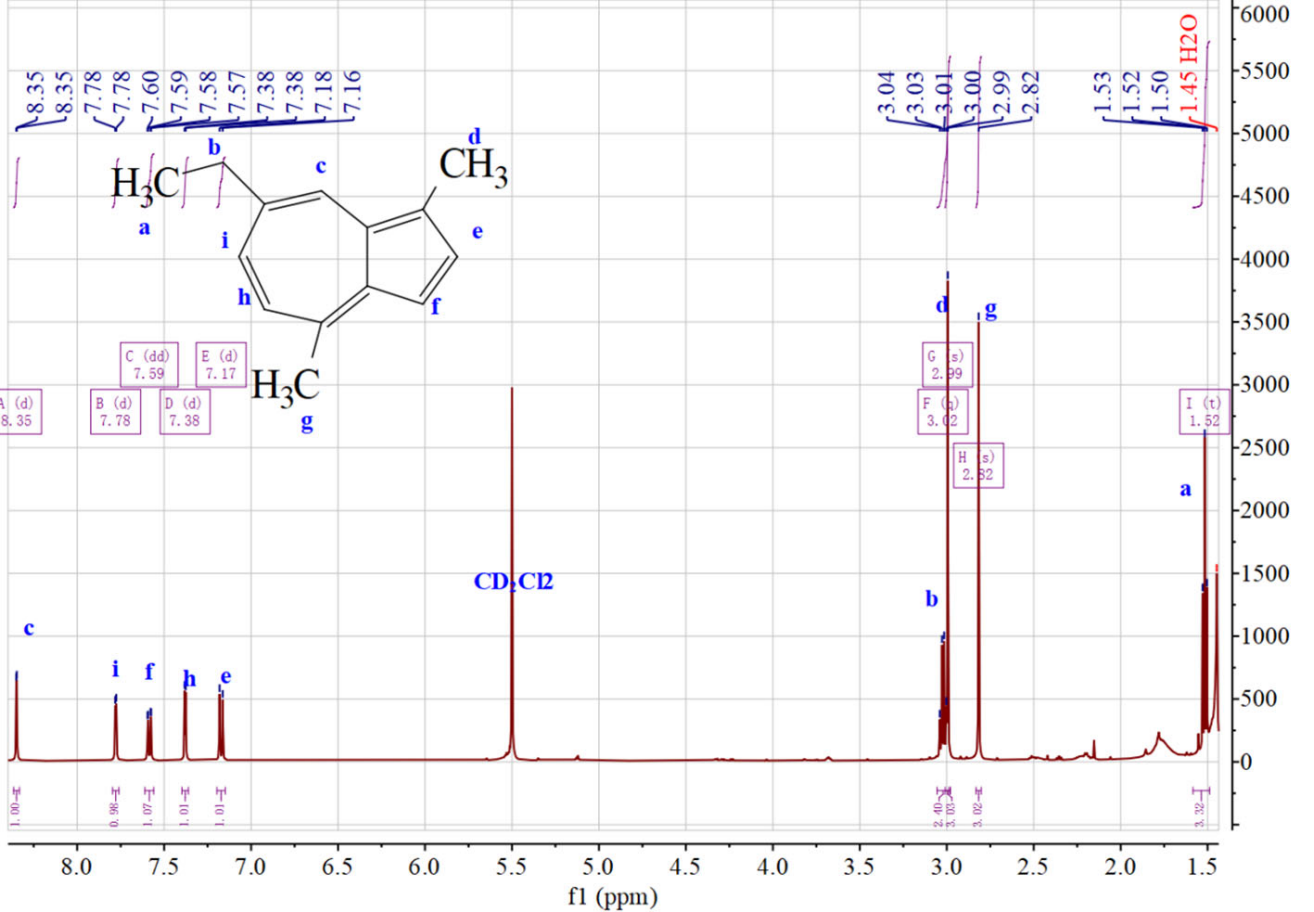
¹H NMR spectrum of chamazulene (600 MHz, CD_2_Cl_2_).

The compound was unequivocally identified as chamazulene based on comprehensive spectroscopic analysis. It appeared as a characteristic blue to bluish-violet oil with the molecular formula C_14_H_16_. The ¹H-NMR spectrum (600 MHz, CD_2_Cl_2_) displayed multiple downfield signals at δH 8.35, 7.78, 7.59, 7.38, 7.17 ppm, corresponding to the highly conjugated azulene framework. In the upfield region, a typical ethyl spin system was identified, consisting of a quartet (δH 3.02, 2H, q, J = 7.6 Hz) coupled to a triplet (δH 1.52, 3H, t, J = 7.6 Hz), along with two isolated methyl singlets at δH 2.99 and 2.82 ppm (each 3H, s) (**Figure 2**).

**Figure 3:**
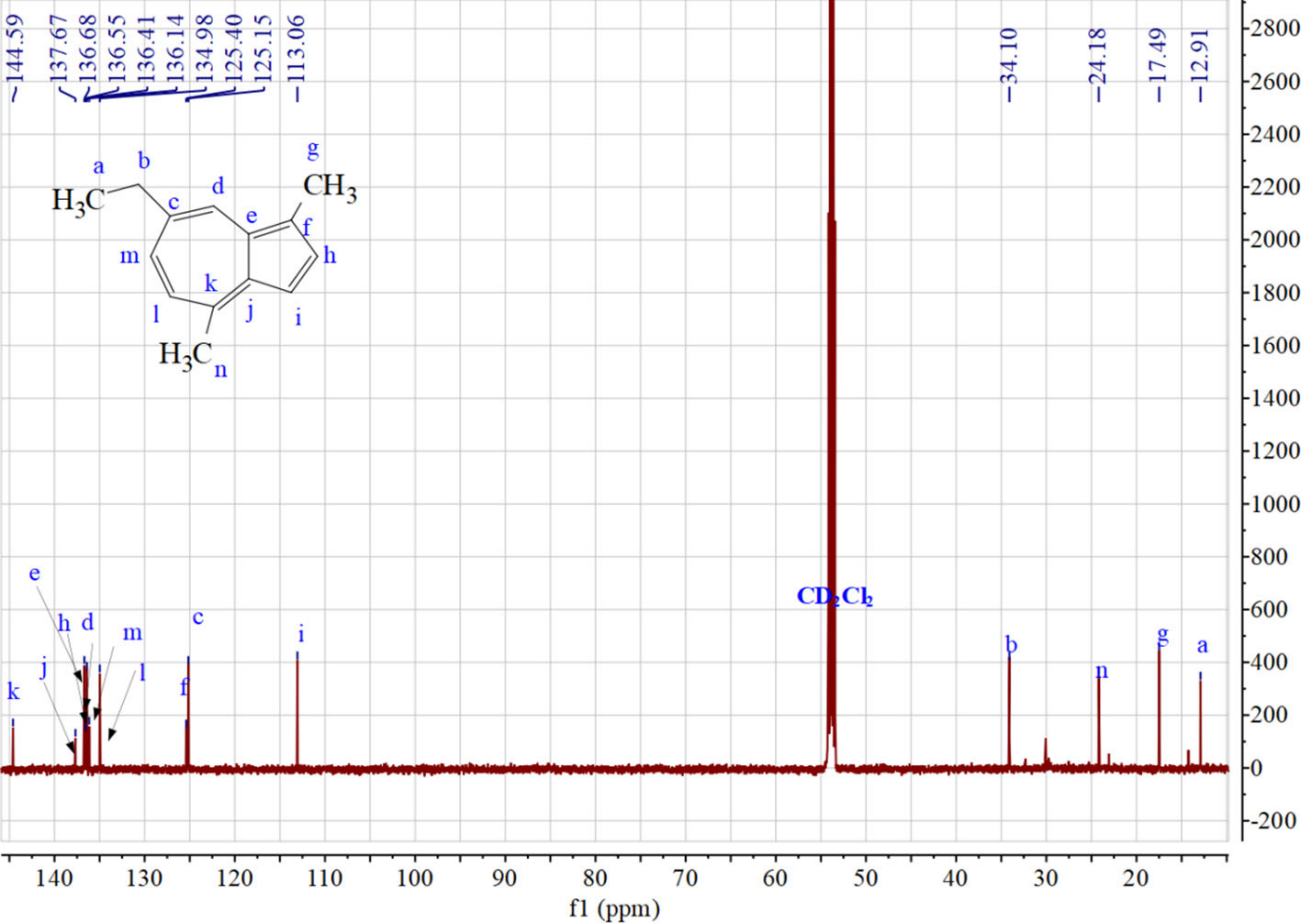
¹³C NMR spectrum of chamazulene (151 MHz, CD_2_Cl_2_).

The ¹³C-NMR spectrum (151 MHz, CD_2_Cl_2_) further supported the structure, showing ten signals attributable to unsaturated carbons of the azulene skeleton (δC 144.59, 137.67, 136.68, 136.55, 136.41, 136.14, 134.98, 125.40, 125.15, 113.06 ppm), along with four signals corresponding to the saturated substituents (δC 34.10, 24.18, 17.49, 12.91 ppm) (**Figure 3**). All NMR data were fully consistent with previously reported spectral characteristics of chamazulene, confirming its chemical identity.

### 3.5 Chemical composition of essential oil

GC–MS analysis identified a total of 36 compounds in the blue essential oil of *A. umbrosa*, accounting for 97.32% of the total composition. The oil was dominated by terpenoids (71.74%) and alcohols (20.65%), with smaller contributions from aromatic compounds (0.99%), phenols (1.36%), ketones (2.18%), and organoboron compounds (0.40%). Chamazulene was the predominant constituent (50.68%), followed by eucalyptol (8.88%), germacrene D (4.05%), 3-cyclohexen-1-ol (3.80%), and caryophyllene oxide (2.59%). Comparative analysis with *A. argyi* yellow essential oil revealed distinct compositional differences, particularly in chamazulene content (**Table 4**).Silica gel treatment exerted minimal influence on the overall chemical profile of the essential oil, with a recovery rate exceeding 96%. Chamazulene content remained stable across treated samples (6.60–6.91%) compared with the untreated control (6.67%). The results from **Table 4** (treatments Tr1–Tr10) show that, in contrast, ketone content decreased markedly from 6.87% in untreated oil to as low as 2.01%, while alcohol content increased from 42.62% to a maximum of 47.49%. These results indicate that silica gel selectively modulates specific odor-active components without substantially altering the major constituents of the oil, supporting its suitability for industrial purification applications (**Table 4**).

### 3.6 Antioxidant activity

**Figure 4:**
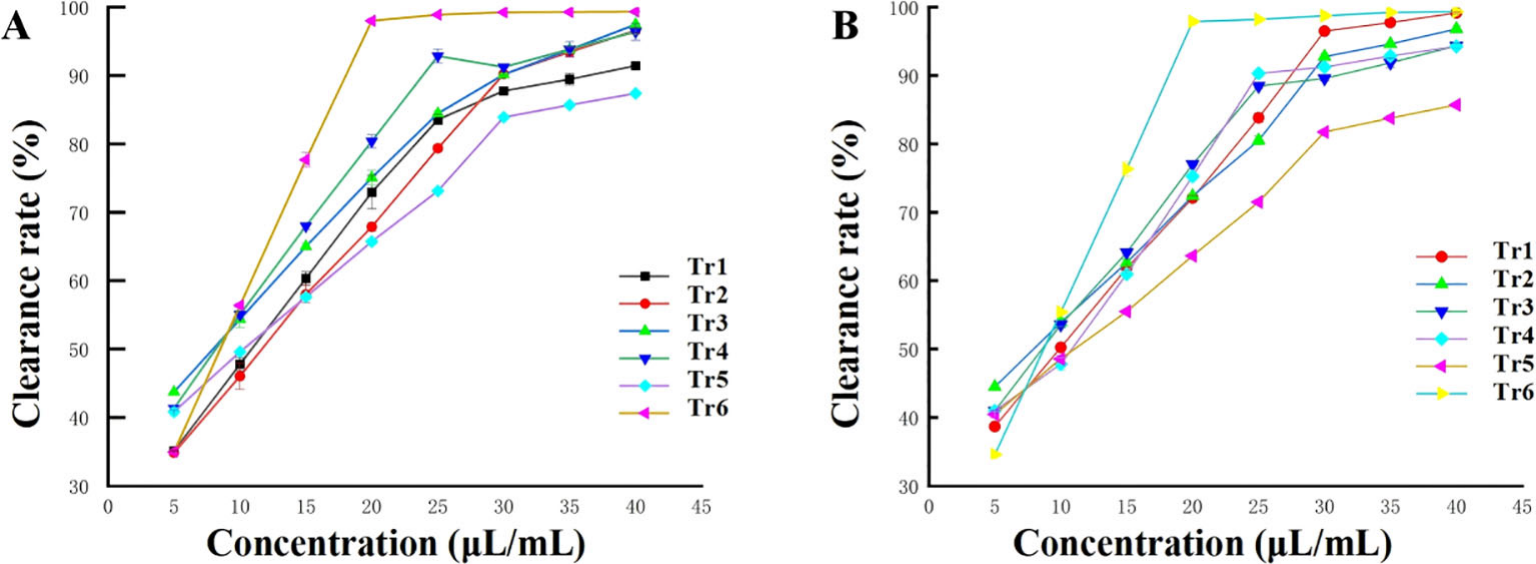
Radical scavenging capacity of essential oils under different treatment conditions and from different species. **(A)** DPPH radical scavenging activity; **(B)** ABTS radical scavenging activity. Tr1, untreated essential oil; Tr2 ~ Tr4, essential oil treated with silica gel at 1 g, 2 g, and 4 g, respectively; Tr5, yellow essential oil from *A. argyi*; Tr6, blue essential oil from *A. umbrosa*.

The blue essential oil exhibited concentration-dependent antioxidant activity in both DPPH and ABTS assays. The IC_50_ values were 8.71 μL/mL for DPPH and 8.86 μL/mL for ABTS, which were significantly lower than those of *A. argyi* yellow essential oil (*p* < 0.05). Following silica gel treatment, antioxidant activity was maintained or slightly enhanced, with IC_50_ values decreasing to 8.03 μL/mL (DPPH) and 8.81 μL/mL (ABTS), indicating that odor modulation did not compromise radical-scavenging capacity (**Figure 4**).

### 3.7 Antimicrobial activity

**Figure 5:**
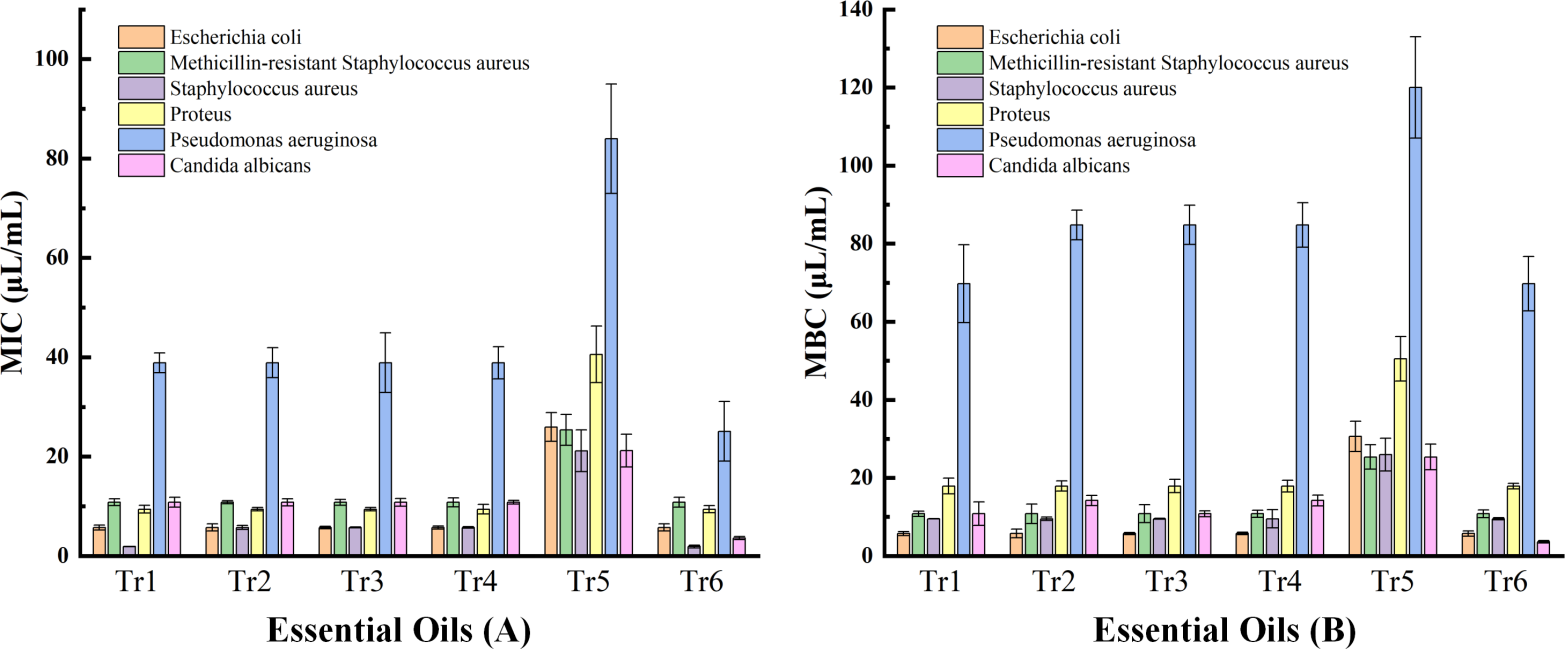
Antimicrobial activity of essential oils under different treatment conditions and different species. **(A)** Minimum inhibitory concentration (MIC); **(B)** Minimum bactericidal concentration (MBC); Tr1, untreated essential oil; Tr2 ~ Tr4, essential oil treated with silica gel at 1 g, 2 g, and 4 g, respectively; Tr5: *A. argyi* yellow essential oil; Tr6: *A. umbrosa* blue essential oil.

The blue essential oil displayed pronounced antimicrobial activity against all tested microorganisms. The lowest MIC values were observed against *Staphylococcus aureus* (21.16 μL/mL) and *Escherichia coli* (25.94 μL/mL), with corresponding MBC values of 25.94 and 30.64 μL/mL, respectively. Inhibitory effects against MRSA and *Candida albicans* were comparatively weaker but remained detectable. Importantly, silica gel-treated oils retained antimicrobial efficacy comparable to that of untreated samples, confirming that sensory optimization did not adversely affect biological activity (**Figure 5**).

### 3.8 Stability evaluation

**Figure 6:**
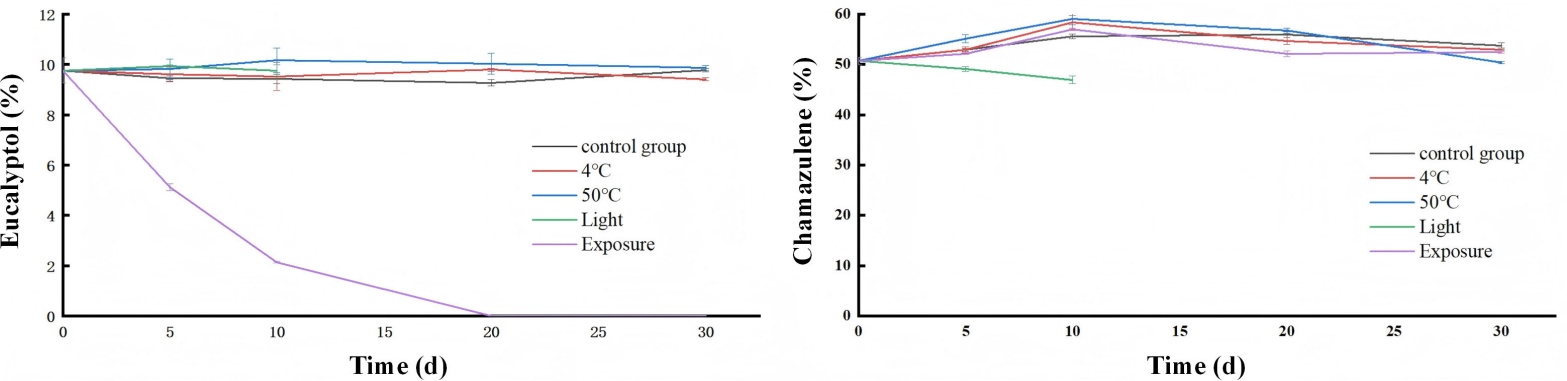
Stability tests of *A. umbrosa* blue essential oil.

As shown in **Figure 6**, thermal stability testing revealed that chamazulene and eucalyptol remained stable at 4 °C, whereas storage at 50 °C resulted in significant degradation, with chamazulene content decreasing by 18.4% after 30 days (*p* < 0.05). Under oxidative conditions, open-air storage led to a 22.7% reduction in chamazulene content, compared with losses of less than 5% under sealed, dark conditions. Photostability experiments showed a 15.6% reduction in chamazulene content after 10 days of light exposure, whereas degradation remained below 3% in darkness (**Supplementary Table 2**). These findings confirm that exposure to heat, oxygen, and light markedly accelerates degradation, highlighting the importance of protective storage conditions.

Samples were placed at 4 °C, 25 °C, and 50 °C under sealed and dark conditions to investigate the effects of heat on the stability of essential oils. Samples were kept in sealed containers under open exposure in a dark environment at room temperature to investigate the effects of oxygen on the stability of essential oils. Samples were subjected to light and dark conditions at room temperature under sealed conditions to investigate the effects of light on the stability of essential oils.

## 4 Discussion

### 4.1 Optimization of UAH extraction and mechanistic implications

The optimized UAH conditions revealed that moderate salinity, a balanced material-to-liquid ratio, and controlled extraction time synergistically enhanced essential oil recovery without compromising thermolabile constituents. These findings are consistent with previous reports on azulene-producing species such as *Matricaria chamomilla* and *Tanacetum annuum*, where ultrasonic cavitation promoted cell wall disruption, accelerated mass transfer, and facilitated the release of azulene precursors while minimizing thermal degradation (Périno-Issartier et al., 2013; Liang et al., 2025).

Salinity emerged as a particularly influential factor in extraction efficiency. The presence of NaCl likely enhanced osmotic pressure gradients between plant tissues and the extraction medium, thereby facilitating the diffusion of intracellular volatiles into the aqueous phase, as reported for salt-assisted essential oil extraction systems (Ghassemi-Golezani and Rahimzadeh, 2022). Importantly, the optimized extraction parameters did not reduce chamazulene content, indicating that the azulene framework remains sufficiently stable under controlled UAH conditions, provided that excessive heating and prolonged extraction are avoided.

### 4.2 Silica gel adsorption as a targeted sensory modulation strategy

A major innovation of this work lies in the application of silica gel adsorption as a post-extraction sensory modulation strategy for blue essential oil. Conventional deodorization approaches, including solvent extraction, molecular distillation, or encapsulation, often result in partial loss of chromatic intensity or bioactive constituents (Irmisch et al., 2012; Jarray et al., 2022). In contrast, silica gel treatment markedly reduced pungent and resinous odor notes while preserving the characteristic blue coloration and maintaining more than 98% chamazulene retention (Tang et al., 2021; Khedhri et al., 2022).

From a flavor chemistry perspective, this effect can be explained by the non-linear relationship between odor perception and the concentration of high-impact odorants (Chen et al., 2023). Sensory intensity is often governed by a limited number of compounds with high odor activity values, particularly ketones and certain phenolic derivatives, which exert disproportionate influence even at low concentrations (Guo et al., 2022). Among these, eucalyptol and (+)-2-bornanone were identified as key odor-active markers in *A. umbrosa* essential oil based on their abundance and documented low odor thresholds (Suleimen et al., 2014). Both compounds possess characteristic camphoraceous, penetrating odor notes and exhibit high vapor pressures, making them readily perceptible even at reduced concentrations. In the present study, silica gel selectively reduced total ketone content while leaving the major terpene matrix largely intact, thereby attenuating irritating odor notes without significantly altering the overall chemical profile. Although direct GC–olfactometry analysis was not performed in the present study, the pronounced reduction in total ketones and phenolic derivatives, widely recognized as high-impact odor contributors, provides a chemically plausible explanation for the observed sensory improvement (Plutowska and Wardencki, 2008). These findings demonstrate a clear structure-odor relationship: the selective adsorption of oxygenated monoterpenes by silica gel effectively modulates the olfactory profile while preserving the characteristic blue color and bioactive chamazulene content.

### 4.3 Structural confirmation: correlation with published NMR data

The experimental ¹H NMR and ¹³C NMR spectra of the isolated compound (refer to the Conclusion section) exhibit remarkable concordance with the documented NMR data for Chamazulene. El Hafidi et al. (2023) identified the characteristic ¹H NMR signals of Chamazulene in the essential oil of Cladanthus scariosus, including aromatic protons of the azulene ring resonating at δH 7.1–8.4 ppm, an ethyl spin system at approximately δH 3.00/1.50, and two aromatic methyl singlets at approximately δH 2.8–3.0 ppm (Rivero-Cruz et al., 2006). Additionally, the ¹³C NMR signals were reported as azulene carbons at δC 110–145 ppm and saturated carbons at δC 12–35 ppm, all of which are consistent with our measured values. Similarly, the NMR data for Chamazulene reported by Rivero-Cruz et al. (2006) in the essential oil of Brickellia veronicaefolia align with our findings. These comparisons substantiate the identification of the isolated compound as Chamazulene (El Hafidi et al., 2023).

The purity of the isolated compound was assessed to be approximately 90% via gas chromatography (GC) analysis; however, no discernible interfering signals were detected in the nuclear magnetic resonance (NMR) spectra. This phenomenon can be attributed to the possibility that when impurity content is below 10% and exhibits limited chemical shift dispersion, the impurity signals are frequently obscured by the predominant peaks (Evans et al., 2026).

### 4.4 Chemical composition and bioactivity: synergistic effects

GC–MS analysis confirmed chamazulene as the predominant constituent (> 50%), accompanied by eucalyptol, germacrene D, and other oxygenated terpenes. The blue essential oil exhibited strong antioxidant activity in both DPPH and ABTS assays, with IC_50_ values lower than those of *A. argyi* yellow essential oil, a widely used reference in traditional and commercial applications. Components frequently present in essential oils, including caryophyllene oxide and caryophyllene, have the potential to augment non-enzymatic antioxidant defenses by elevating the levels of reduced glutathione (GSH) (Baldissera et al., 2017). These findings align with prior research that attributes significant radical-scavenging capabilities to azulene derivatives and oxygenated monoterpenes (Ornano et al., 2013; El Mihyaoui et al., 2022). This consistency may be attributed to chamazulene’s antioxidant mechanism, which involves the reduction of lipid peroxidation and the enhancement of the activities of the enzymes superoxide dismutase (SOD), glutathione peroxidase (GPx), and catalase (CAT) (Ma et al., 2020). Consequently, these results underscore the functional potential of *A. umbrosa* blue oil (Aati et al., 2024).

The chemical composition and bioactivity of *A. umbrosa* essential oil have been scarcely reported. A notable previous study by Suleimen et al. analyzed oil from Kazakh *A. umbrosa*, identifying chamazulene (35.7%) as major constituents, and reported antimicrobial activity (Aleksic Sabo and Knezevic, 2019). The pronounced antimicrobial activity, particularly against *Staphylococcus aureus* and *Escherichia coli*, likely arises from synergistic interactions among sesquiterpenes and oxygenated monoterpenes, which disrupt microbial membranes and interfere with cellular metabolism (da Silva Moura et al., 2020; Mączka et al., 2021). Although chamazulene is the predominant constituent, the antioxidant and antimicrobial activities observed here cannot be attributed to a single compound alone but are more likely driven by synergistic interactions among multiple terpenoid components (Hummels et al., 2023; Nasrollahian et al., 2024). Caryophyllene, a compound frequently present in essential oils, has been documented to inhibit bacterial efflux pumps and induce non-selective pore formation within the bacterial membrane (Bencze et al., 2023). Notably, silica gel–treated oils retained comparable bioactivities to untreated samples, indicating that odor-active components are not the primary determinants of biological efficacy.

### 4.5 Stability considerations and implications for storage

The observed degradation of chamazulene under heat, light, and oxidative conditions is consistent with previous reports on the instability of azulene-type sesquiterpenes in essential oils (Hojerová et al., 2011). Similar sensitivity to thermal and photo-oxidative degradation has been documented for chamazulene-rich oils from *Matricaria recutita* L. and *Achillea millefolium* L. Gabbanini et al. (2024). recently demonstrated that chamazulene in *Artemisia*, *Matricaria*, and *Achillea* essential oils undergoes rapid photochemical and oxidative degradation, which aligns closely with our findings. Furthermore, the protective role of appropriate storage conditions against photodegradation has been emphasized in studies of photosensitive cosmetic ingredients. These findings confirm that exposure to heat, oxygen, and light markedly accelerates degradation, highlighting the importance of protective storage conditions.

### 4.6 Overall significance and future perspectives

This study identifies *A. umbrosa* as a promising source of chamazulene-rich blue essential oil, addressing critical challenges in extraction efficiency and odor mitigation through an integrated approach that combines ultrasound-assisted hydrodistillation optimization with silica gel adsorption. The application of response surface methodology ensures methodological rigor, while the quantitative monitoring of eucalyptol and (+)-2-bornanone provides an objective measure of deodorization efficacy. Complementary bioactivity and stability profiling further substantiate its potential for industrial and formulation applications. However, limitations such as the lack of chemotypic diversity, the absence of gas chromatography-olfactometry, and the necessity for scale-up and techno-economic validation must be acknowledged. In addition, we were unable to perform expanded stability studies to evaluate changes in overall antioxidant activity, antimicrobial activity, and color retention after storage. Future research should systematically assess these bioactivity and color stability parameters under different storage conditions (e.g., temperature, light, air exposure). Future research should focus on mechanistic elucidation using omics tools, exploration of the roles of minor constituents, and the development of advanced delivery systems to enhance stability and functionality, thereby reinforcing the commercial viability of this essential oil in the natural product sectors.

## 5 Conclusion

This study provides an integrated framework for the efficient production and quality optimization of chamazulene-rich blue essential oil from *A. umbrosa*, addressing two long-standing bottlenecks that have constrained the industrial utilization of blue essential oils: limited extraction efficiency and poor sensory acceptability. By coupling UAH with RSM and introducing a silica gel-based post-extraction deodorization strategy, we demonstrate that yield enhancement, odor modulation, and bioactivity preservation can be simultaneously achieved within a single processing chain.

## Data Availability

The original contributions presented in the study are included in the article/**Supplementary Material**. Further inquiries can be directed to the corresponding author.

## Supplementary material

Supplementary Table 1 Results of one-way experiments of blue essential oil of *Artemisia umbrosa.*

Supplementary Table 2 Compositional analysis of *Artemisia umbrosa* blue essential oil stability study.

Supplementary Figure 1 Chromatographic analysis of chamazulene isolation and purification. **(A)** Chromatogram of chamazulene standard; **(B)** Chromatogram of chamazulene from A. umbrosa oil.

Supplementary Figure 2 Radical scavenging capacity of essential oils under different treatment conditions and from different species. **(A)** DPPH radical scavenging activity; **(B)** ABTS radical scavenging activity.
