# Supplementary material for: Optimized extraction, odor modulation, and antioxidant and antimicrobial activities of blue essential oil from Artemisia umbrosa
Source: Front Plant Sci. 2026 Jun 10;17:1826250. doi: 10.3389/fpls.2026.1826250 (PMC13290947; doi:10.3389/fpls.2026.1826250)
Supplement: Supplementary Table 1 — Results of one-way experiments of blue essential oil of Artemisia umbrosa. [file Table1.docx]

**Table S1** Results of one-way experiments of blue essential oil of *Artemisia umbrosa*

| Factor | Treatment | Extraction rate (M ± SD%) |
| --- | --- | --- |
| Drying method | Dried | 0.64 ± 0.00 |
|  | Fresh | 0.67 ± 0.04 |
| Degree of pulverization | 20 mesh | 0.93 ± 0.03 |
|  | 40 mesh | 0.95 ± 0.03 |
|  | Blade | 1.14 ± 0.05 |
| Salinity | 0% | 0.99 ± 0.03 |
|  | 2% | 1.14 ± 0.05 |
|  | 3% | 1.18 ± 0.09 |
|  | 4% | 1.00 ± 0.03 |
| Material-liquid ratio | 1:8 | 1.14 ± 0.05 |
|  | 1:10 | 1.22 ± 0.04 |
|  | 1:12 | 1.35 ± 0.07 |
|  | 1:14 | 1.46 ± 0.05 |
|  | 1:16 | 1.30 ± 0.09 |
| Ultrasound time | 0 min | 1.25 ± 0.01 |
|  | 15 min | 1.28 ± 0.00 |
|  | 30 min | 1.14 ± 0.05 |
|  | 45 min | 1.11 ± 0.05 |
| Extraction time | 2 h | 1.14 ± 0.05 |
|  | 3 h | 1.26 ± 0.02 |
|  | 4 h | 1.40 ± 0.06 |

Note: The extraction rate for shade drying is calculated using the weight of fresh leaves before drying, if calculated on the basis of the mass after drying then the extraction rate is (1.21 ± 0.04)%. Data are reported as the M ± SD% (*n* = 3).
