# Supplementary material for: Optimized extraction, odor modulation, and antioxidant and antimicrobial activities of blue essential oil from Artemisia umbrosa
Source: Front Plant Sci. 2026 Jun 10;17:1826250. doi: 10.3389/fpls.2026.1826250 (PMC13290947; doi:10.3389/fpls.2026.1826250)
Supplement: Supplementary Table 2 — Compositional analysis of Artemisia umbrosa blue essential oil stability study. [file Table2.docx]

**Table S2** Compositional analysis of *Artemisia umbrosa* blue essential oil stability study

| No. | RT | RI | Compounds | Molecular formula | Molecular weight | Relative content (M ± SD%) | | | | | | | | | | | | | | | |
| --- | --- | --- | --- | --- | --- | --- | --- | --- | --- | --- | --- | --- | --- | --- | --- | --- | --- | --- | --- | --- | --- |
|  |  |  |  |  |  | Y-0 | YL-5 | YL-10 | YC-10 | YC-20 | YC-30 | YO-10 | YO-20 | YO-30 | YH-10 | YH-20 | YH--30 | Y-5 | Y-10 | Y-20 | Y-30 |
| 1 | 7.78 | 1098.5 | 1,8-Cineole | C_10_H_16_ | 136 | 0.57±0.03 | 0.61±0.06 | 0.51±0.10 | 0.61±0.00 | 0.61±0.04 | 0.60±0.00 | - | - | - | - | - | - | 0.59±0.01 | 0.58±0.01 | 0.61±0.05 | 0.75±0.00 |
| 2 | 8.27 | 990.8 | *β*-Myrcene | C_10_H_16_ | 136 | 1.00±0.05 | 2.29±0.18 | 1.93±0.37 | 1.87±0.16 | 2.15±0.14 | 2.32±0.06 | - | - | - | 1.90±0.09 | 1.98±0.14 | 2.21±0.08 | 2.10±0.02 | 2.19±0.06 | 2.14±0.19 | 2.74±0.05 |
| 3 | 8.63 | 1025.7 | *α*-Phellandrene | C_10_H_16_ | 136 | 0.36±0.02 | - | - | - | - | - | - | - | - | - | - | - | - | - | - | - |
| 4 | 8.98 | 1053.5 | *p*-*α*-Phellandrene | C_10_H_16_ | 136 | 1.01±0.05 | 0.46±0.04 | 0.33±0.23 | - | - | - | - | - | - | - | - | - | - | - | - | 0.61±0.02 |
| 5 | 9.13 | 1066.9 | D-limonene | C_10_H_16_ | 136 | 0.46±0.02 | - | - | - | - | - | - | - | - | - | - | - | - | - | - | 0.49±0.01 |
| 6 | 9.21 | 1058.6 | *o*-Cymene | C_10_H_14_ | 134 | 0.44±0.02 | - | - | - | - | - | - | - | - | - | - | - | - | - | - | - |
| 7 | 9.40 | 1032.0 | Eucalyptol | C_10_H_18_O | 154 | 8.88±0.49 | 9.93±0.06 | 9.76±0.22 | 9.52±0.55 | 9.79±0.06 | 9.41±0.05 | 2.14±0.03 | - | - | 10.17±0.68 | 10.03±0.52 | 9.86±0.10 | 9.44±0.10 | 9.43±0.19 | 9.27±0.13 | 9.77±0.07 |
| 8 | 10.20 | 1059.7 | *γ*-Terpinene | C_10_H_16_ | 136 | 1.66±0.08 | 0.93±0.08 | 0.84±0.16 | 0.83±0.05 | 0.85±0.03 | 0.99±0.01 | - | - | - | 0.81±0.01 | 0.81±0.08 | 1.01±0.02 | 0.87±0.01 | 0.87±0.03 | 0.82±0.05 | 0.74±0.04 |
| 9 | 10.45 | 1074.9 | Sabinol | C_10_H_18_O | 154 | - | 2.32±0.24 | 1.79±0.35 | 1.91±0.13 | 2.00±0.05 | 2.20±0.01 | 2.00±0.03 | 1.73±0.05 | 0.60±0.02 | 1.84±0.08 | 1.96±0.09 | 2.05±0.02 | 2.13±0.06 | 2.09±0.04 | 1.77±0.11 | 1.64±0.01 |
| 10 | 11.05 | 1134.7 | *p*-Mentha-1,3-diene | C_10_H_16_ | 136 | 0.37±0.00 | - | - | - | - | - | - | - | - | - | - | - | - | - | - | - |
| 11 | 11.35 | 1154.8 | (–)-Borneol | C_10_H_18_O | 154 | - | 0.74±0.06 | 0.61±0.12 | - | 0.65±0.06 | 0.71±0.01 | 0.77±0.01 | 1.02±0.17 | - | - | - | 0.70±0.00 | 0.63±0.01 | 0.64±0.02 | 0.60±0.05 | 0.59±0.02 |
| 12 | 11.40 | 1098.9 | Linalool | C_10_H_18_O | 154 | 0.28±0.10 | - | - | - | - | - | - | - | - | - | - | - | - | - | - | - |
| 13 | 11.50 | 1107.8 | *α*-Vetivone | C_10_H_14_O | 150 | 0.30±0.02 | 0.62±0.05 | 0.52±0.11 | 0.58±0.00 | - | 0.65±0.01 | - | - | - | - | 0.19±0.00 | 0.64±0.01 | 0.18±0.25 | 0.19±0.27 | - | - |
| 14 | 12.66 | 1143.9 | (+)-2-Bornanone | C_10_H_16_O | 152 | 1.88±0.08 | 2.42±0.28 | 2.15±0.45 | 2.28±0.03 | 2.24±0.07 | 2.55±0.03 | 2.36±0.07 | 2.02±0.06 | 0.86±0.02 | 2.38±0.09 | 2.41±0.12 | 2.57±0.09 | 2.34±0.01 | 2.39±0.03 | 2.22±0.14 | 2.20±0.04 |
| 15 | 13.30 | 1315.7 | endo-Borneol | C10H18O | 154 | 0.76±0.03 | 0.72±0.07 | 0.44±0.31 | 0.57±0.00 | 0.59±0.03 | 0.66±0.01 | 0.87±0.02 | 1.28±0.24 | 0.77±0.01 | 0.20±0.01 | - | 0.66±0.00 | 0.62±0.01 | 0.59±0.02 | 0.59±0.02 | 0.55±0.04 |
| 16 | 13.63 | 1346.8 | *(R)*-(–)-*α*-Terpineol | C_10_H_18_O | 154 | 3.80±0.09 | 2.78±0.26 | 2.51±0.48 | 2.42±0.11 | 2.42±0.09 | 3.01±0.03 | 3.32±0.07 | 3.27±0.25 | 2.34±0.00 | 2.52±0.02 | 2.53±0.07 | 3.01±0.02 | 2.61±0.10 | 2.60±0.11 | 2.60±0.12 | 2.36±0.03 |
| 17 | 14.02 | 1382.6 | *α*-Terpineol | C_10_H_18_O | 154 | 1.57±0.07 | 1.90±0.15 | 1.60±0.26 | 1.24±0.05 | 1.50±0.09 | 1.82±0.13 | 2.37±0.05 | 3.30±0.16 | 2.43±0.02 | 1.24±0.04 | 1.31±0.06 | 1.83±0.10 | 1.69±0.11 | 1.67±0.08 | 1.69±0.12 | 1.67±0.03 |
| 18 | 14.82 | 1457.9 | trans-Carveol | C_10_H_18_O | 154 | - | - | - | - | - | - | - | 0.72±0.01 | 0.58±0.01 | - | - | - | - | - | - | - |
| 19 | 16.32 | 1620.5 | Saffranal | C_10_H_14_O | 150 | - | - | - | - | - | - | - | 0.54±0.02 | - | - | - | - | - | - | - | - |
| 20 | 19.41 | 1417.7 | Caryophyllene | C_15_H_24_ | 204 | 2.59±0.12 | 1.84±0.20 | 1.75±0.06 | 1.65±0.01 | 1.67±0.03 | 1.93±0.00 | 2.45±0.03 | 2.79±0.16 | 2.68±0.03 | 1.74±0.03 | 1.67±0.06 | 1.92±0.02 | 1.75±0.02 | 1.80±0.02 | 1.64±0.09 | 1.65±0.04 |
| 21 | 19.98 | 1517.9 | Humulene | C_15_H_24_ | 204 | 0.78±0.02 | - | - | - | - | - | - | - | - | - | - | - | - | - | - | - |
| 22 | 20.19 | 1537.8 | (–)-cis-Rose oxide | C_15_H_24_O | 220 | 0.50±0.06 | 0.52±0.37 | - | - | - | - | - | - | - | 3.33±0.07 | 2.83±0.03 | - | - | - | - | - |
| 23 | 20.43 | 1480.8 | Germacrene D | C_15_H_24_ | 204 | 4.05±0.14 | 3.81±0.03 | 3.40±0.19 | 3.32±0.06 | 3.35±0.09 | 3.89±0.00 | 4.91±0.05 | 5.79±0.08 | 5.69±0.04 | - | - | 2.88±0.04 | 3.48±0.02 | 3.60±0.09 | 3.35±0.18 | 3.22±0.04 |
| 24 | 20.51 | 1577.9 | *β*-Caryophyllene | C_15_H_24_ | 204 | 1.47±0.07 | 1.86±0.16 | 1.08±0.76 | 1.66±0.04 | 1.59±0.05 | 1.78±0.00 | 2.19±0.03 | 2.55±0.22 | 2.60±0.01 | - | 1.74±0.12 | 2.48±0.02 | 1.62±0.01 | 1.67±0.04 | 1.59±0.07 | 1.57±0.01 |
| 25 | 20.66 | 1591.7 | Cyclosativene | C_15_H_24_ | 204 | 1.49±0.10 | 1.59±0.21 | 1.39±0.32 | 1.74±0.01 | 1.61±0.05 | 1.76±0.01 | 2.17±0.01 | 2.72±0.10 | 2.54±0.01 | - | 1.66±0.03 | 1.74±0.01 | 1.67±0.00 | 1.69±0.03 | 1.62±0.09 | 1.67±0.02 |
| 26 | 21.84 | 1698.6 | Guaiol | C_15_H_26_O | 222 | 1.88±0.08 | 3.37±0.30 | 2.99±0.57 | 2.94±0.05 | 3.08±0.04 | 3.50±0.00 | 4.59±0.04 | 5.52±0.37 | 5.96±0.03 | 3.03±0.05 | 3.06±0.08 | 3.42±0.03 | 3.23±0.05 | 3.30±0.12 | 3.08±0.19 | 3.96±0.04 |
| 27 | 21.92 | 1579.9 | Caryophyllene oxide | C_15_H_24_O | 220 | 1.22±0.09 | 1.56±0.16 | 1.39±0.30 | 1.44±0.02 | 1.42±0.05 | 1.57±0.01 | 2.16±0.04 | 2.59±0.18 | 2.77±0.01 | 1.44±0.03 | 1.45±0.04 | 1.54±0.02 | 1.52±0.02 | 1.54±0.04 | 1.39±0.06 | 1.82±0.01 |
| 28 | 22.29 | 1550.8 | Diepicedrene-1-oxide | C_15_H_24_O | 220 | 0.36±0.07 | 0.32±0.23 | 0.53±0.46 | - | - | - | 0.65±0.01 | - | - | - | - | - | - | - | - | - |
| 29 | 22.36 | 1518.9 | 3,6-Dihydrochamazulene | C_14_H_18_ | 186 | 0.63±0.09 | - | - | - | - | - | - | 0.73±0.27 | - | - | - | - | - | - | - | - |
| 30 | 22.40 | 1812.6 | Bacdanol | C_20_H_38_O_2_ | 310 | - | 0.14±0.01 | - | - | - | - | - | - | - | - | - | - | - | - | - | - |
| 31 | 22.54 | 1749.7 | 1-phenyl-2-(2,2,3,3-tetramethylcyclopropylidene)ethene | C_14_H_18_ | 186 | 0.55±0.03 | - | - | - | - | - | - | 0.27±0.38 | - | - | - | - | - | - | - | - |
| 32 | 22.63 | 1576.9 | (-)-Spathulenol | C_15_H_26_O | 222 | 0.49±0.03 | 0.38±0.27 | 0.80±0.37 | - | - | - | 0.82±0.02 | 1.10±0.05 | 1.25±0.01 | - | - | - | - | - | - | - |
| 33 | 22.67 | 1760.5 | tau.-Muurolol | C_15_H_26_O | 222 | - | 0.43±0.30 | 0.64±0.10 | 0.68±0.06 | 0.61±0.07 | 0.77±0.07 | 0.97±0.12 | 1.18±0.05 | 1.16±0.00 | 0.67±0.03 | 0.66±0.06 | 0.84±0.00 | 0.71±0.08 | 0.71±0.10 | 0.56±0.02 | 0.75±0.01 |
| 34 | 22.86 | 1659.8 | Neointermedeol | C_15_H_26_O | 222 | 2.06±0.10 | 1.65±0.32 | 1.50±0.28 | 1.29±0.04 | 1.22±0.13 | 1.39±0.08 | 1.79±0.04 | 1.65±0.61 | 2.46±0.01 | 1.11±0.01 | 1.17±0.07 | 1.45±0.10 | 1.36±0.11 | 1.30±0.06 | 1.12±0.06 | 1.06±0.06 |
| 35 | 23.02 | 1795.1 | Ledene oxide-(II) | C_15_H_24_O | 220 | 0.55±0.08 | - | - | - | 0.64±0.00 | 0.21±0.29 | - | - | - | - | - | - | - | - | - | - |
| 36 | 23.15 | 1806.8 | *α*-Bisabolol | C_15_H_26_O | 222 | 0.57±0.06 | - | - | - | - | - | - | - | - | - | - | - | - | - | - | - |
| 37 | 23.23 | 1814.9 | Humulenol-II | C_15_H_24_O | 220 | 0.56±0.05 | - | - | - | - | - | - | 1.11±0.07 | - | - | - | - | - | - | - | - |
| 38 | 23.77 | 1724.7 | Chamazulene | C_14_H_16_ | 184 | 50.68±0.72 | 49.11±0.44 | 46.93±0.78 | 58.33±1.43 | 54.68±0.69 | 52.96±0.12 | 56.96±0.19 | 52.09±0.56 | 52.41±0.15 | 59.09±1.21 | 56.68±0.56 | 53.36±0.25 | 52.90±0.30 | 55.61±0.48 | 55.96±0.59 | 53.78±0.51 |
| 39 | 24.19 | 1859.8 | (+)-Nootkatol | C_15_H_24_O | 220 | 0.36±0.02 | 0.82±0.02 | 0.50±0.35 | - | - | 0.65±0.07 | 1.07±0.02 | 0.60±0.12 | 1.50±0.05 | - | - | 0.72±0.02 | 0.79±0.01 | 0.71±0.05 | 1.00±0.03 | 0.92±0.01 |
| 40 | 24.32 | 1784.9 | Dehydrochamazulene | C_14_H_14_ | 182 | 1.43±0.18 | 1.86±0.19 | 1.67±0.35 | 1.40±0.04 | 1.82±0.12 | 1.85±0.12 | 2.66±0.04 | - | 2.94±0.03 | 1.32±0.03 | 1.43±0.25 | 1.85±0.02 | 1.89±0.05 | 1.83±0.08 | - | 2.42±0.02 |
| 41 | 24.42 | 1885.4 | Cyclohexasiloxane, octadecamethyl- | C_12_H_36_O_6_Si_6_ | 444 | - | 1.03±0.56 | 2.64±1.44 | - | - | - | - | 0.78±0.25 | - | - | - | - | - | - | - | - |
| 42 | 24.67 | 1902.6 | Cycloheptasiloxane,tetradecamethyl- | C_14_H_42_O_7_Si_7_ | 518 | - | 1.71±0.60 | 6.12±2.04 | - | - | - | - | 0.80±0.21 | - | - | - | - | - | 0.79±1.11 | - | - |
| 43 | 26.16 | 2018.3 | Octasiloxane,1,1,3,3,5,5,7,7,9,9,11,11,13,13,15,15-hexadecamethyl- | C_15_H_50_O_7_Si_8_ | 578 | - | - | 0.79±0.38 | - | - | - | - | 0.10±0.14 | - | - | - | - | - | - | - | - |
| 44 | 27.53 | 2120.5 | Phytol | C_20_H_40_O | 296 | - | - | - | - | - | - | - | - | 0.36±0.25 | - | - | - | - | - | - | - |
| **Alcohols** | | | | | | 20.65 | 25.18 | 23.14 | 20.57 | 21.86 | 24.12 | 20.71 | 21.37 | 19.41 | 20.78 | 20.72 | 24.54 | 23.21 | 23.04 | 22.28 | 23.27 |
| **Aromatic** | | | | | | 0.55 | - | - | - | - | - | - | 0.27 | - | - | - | - | - | - | - | - |
| **Phenols** | | | | | | 1.36 | 1.17 | 1.34 | 3.64 | 3.21 | 1.49 | - | 0.25 | 0.57 | 3.20 | 4.75 | 1.48 | 4.00 | 0.93 | 2.51 | 0.48 |
| **Siloxanes** | | | | | | - | 2.74 | 9.55 | - | - | - | - | 1.72 | - | - | - | - | - | 0.79 | - | - |
| **Ethers** | | | | | | 0.50 | 0.52 | - | - | - | - | - | - | - | 3.33 | 2.83 | - | - | - | - | - |
| **Aldehydes** | | | | | | - | - | - | - | - | - | - | 0.54 | - | - | - | - | - | - | - | - |
| **Terpenes** | | | | | | 71.22 | 66.24 | 61.75 | 72.85 | 70.39 | 69.86 | 74.15 | 70.37 | 71.63 | 66.30 | 67.42 | 68.99 | 68.39 | 71.38 | 69.12 | 70.97 |
| **Alkenes** | | | | | | 0.46 | - | - | - | - | - | - | - | - | - | - | - | - | - | - | 0.49 |
| **Ketones** | | | | | | 2.18 | 3.04 | 2.67 | 2.86 | 2.24 | 3.20 | 2.36 | 2.02 | 0.86 | 2.38 | 2.60 | 3.21 | 2.52 | 2.58 | 2.22 | 2.20 |
| **Total** | | | | | | 95.56 | 97.72 | 97.11 | 96.28 | 94.49 | 97.18 | 97.22 | 96.29 | 91.90 | 92.79 | 93.57 | 96.82 | 94.12 | 97.79 | 93.62 | 96.93 |

Note: Y-0, Y-5, Y-10, Y-20, Y-30, Store at room temperature in a sealed container for 0 d, 5 d, 10 d, 20 d, 30 d, the control group corresponding to Figure 6; YL-5, YL-10, Store under light exposure for 5 d, 10 d; YC-10,The light group corresponding to Figure 6; YC-20, YC-30, Store at 4℃ in a sealed container for 10 d, 20 d, 30 d, The 4℃ group corresponding to Figure 6; YO-10, YO-20, YO-30, Store at room temperature in an open container for 10 d, 20 d, 30 d, The exposure group corresponding to Figure 6; YH-10, YH-20, YH-30, Store at 50℃ in a sealed condition for 10 d, 20 d, 30 d, The 50℃ group corresponding to Figure 6. Data are reported as the M ± SD% (n = 3). Data are reported as the M ± SD% (*n* = 3). Experimental linear retention indices (RI) were calculated against a C₇–C₃₀ n-alkane mix and matched with NIST 17 Chemistry WebBook (± 5-unit tolerance); The table lists the Common Name (or the Systematic Name if no established common name exists) corresponding to each provided systematic chemical name.
