# Supplementary figures and images for: Optimized extraction, odor modulation, and antioxidant and antimicrobial activities of blue essential oil from Artemisia umbrosa
Source: Front Plant Sci. 2026 Jun 10;17:1826250. doi: 10.3389/fpls.2026.1826250 (PMC13290947; doi:10.3389/fpls.2026.1826250)

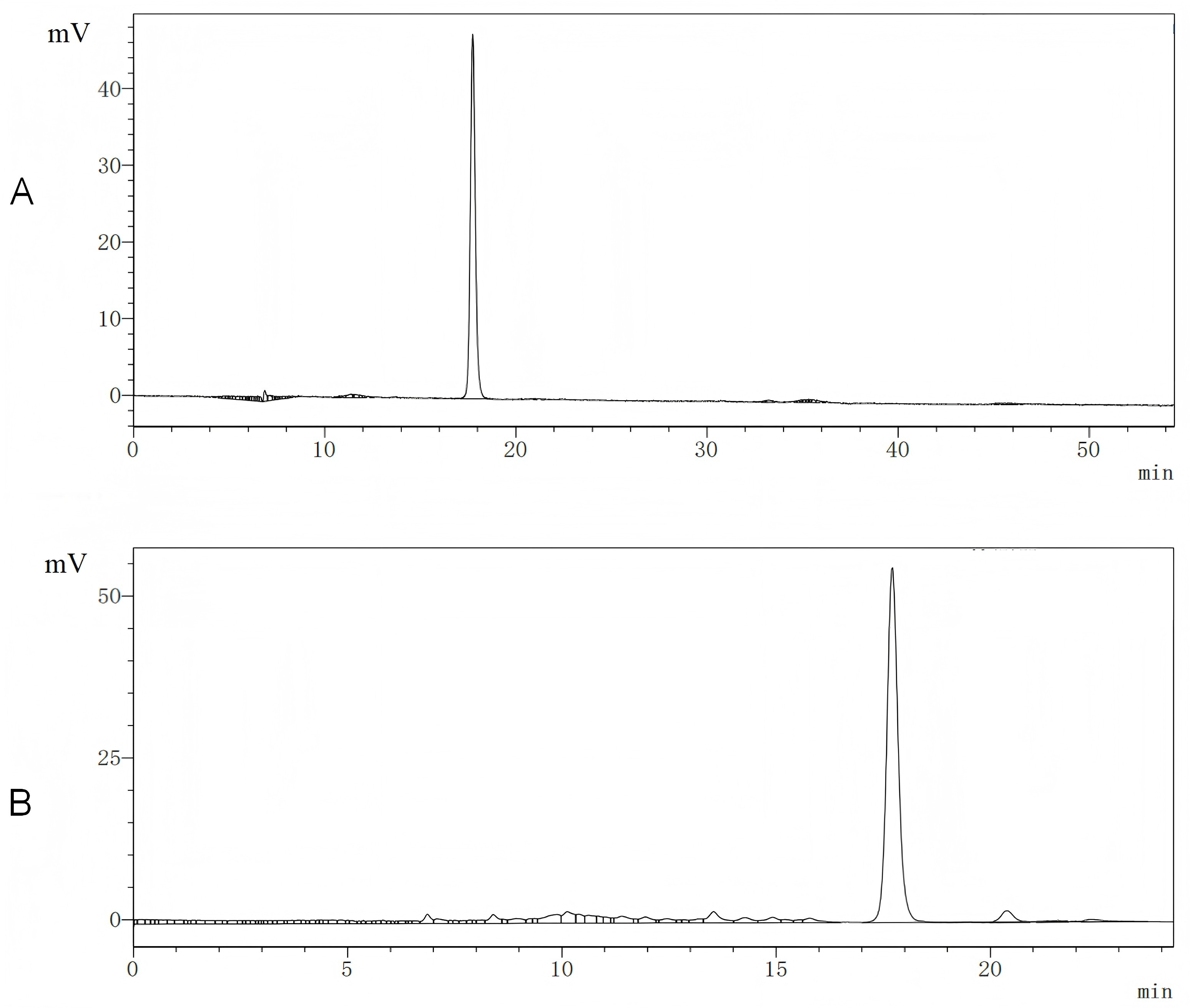

Supplement: Supplementary Figure 1 — Chromatographic analysis of chamazulene isolation and purification. (A) Chromatogram of chamazulene standard; (B) Chromatogram of chamazulene from A. umbrosa oil. [file Image1.tiff]

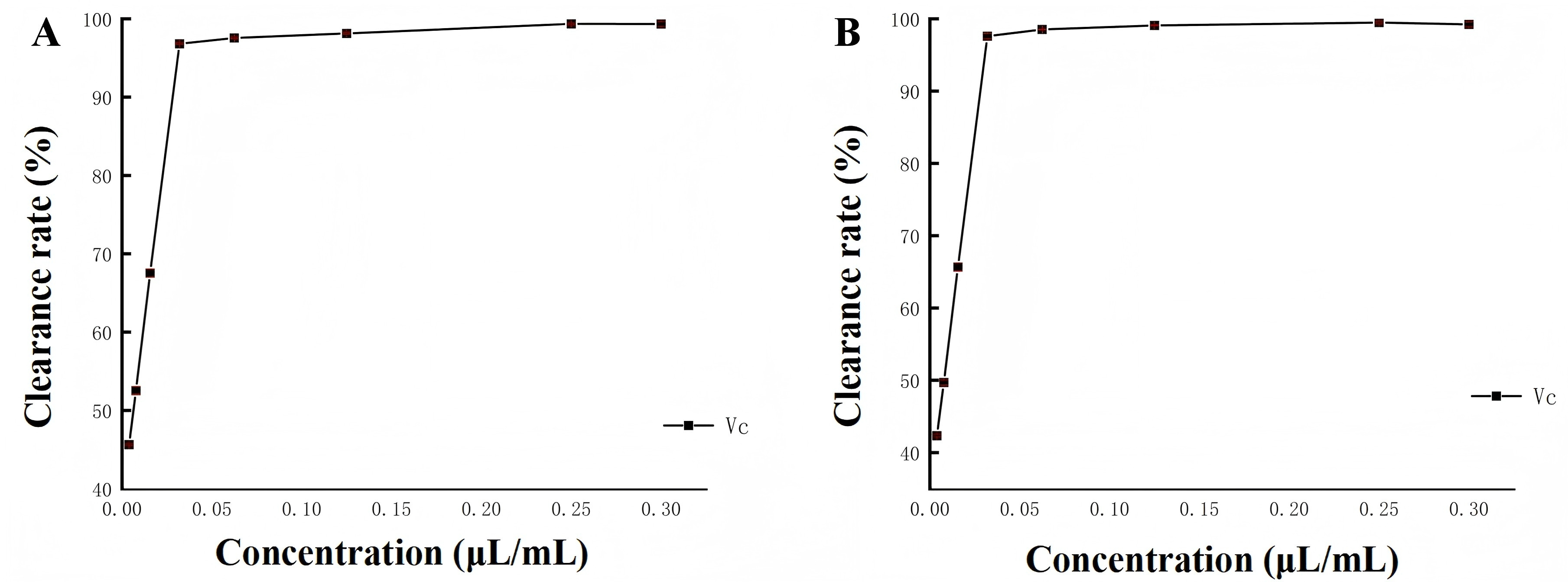

Supplement: Supplementary Figure 2 — Radical scavenging capacity of essential oils under different treatment conditions and from different species. (A) DPPH radical scavenging activity; (B) ABTS radical scavenging activity. [file Image2.tiff]
